# Polyamines Promote Aragonite Nucleation and Generate Biomimetic Structures

**DOI:** 10.1002/advs.202203759

**Published:** 2022-11-20

**Authors:** Ouassef Nahi, Alexander N. Kulak, Shuheng Zhang, Xuefeng He, Zabeada Aslam, Martha A. Ilett, Ian J. Ford, Robert Darkins, Fiona C. Meldrum

**Affiliations:** ^1^ School of Chemistry University of Leeds Woodhouse Lane Leeds LS2 9JT UK; ^2^ School of Chemical and Process Engineering University of Leeds Woodhouse Lane Leeds LS2 9JT UK; ^3^ London Centre for Nanotechnology University College London 17–19 Gordon Street London WC1H 0AH UK

**Keywords:** biomineralization, calcium carbonate, magnesium, non‐classical crystallization, polymorph

## Abstract

Calcium carbonate biomineralization is remarkable for the ability of organisms to produce calcite or aragonite with perfect fidelity, where this is commonly attributed to specific anionic biomacromolecules. However, it is proven difficult to mimic this behavior using synthetic or biogenic anionic organic molecules. Here, it is shown that cationic polyamines ranging from small molecules to large polyelectrolytes can exert exceptional control over calcium carbonate polymorph, promoting aragonite nucleation at extremely low concentrations but suppressing its growth at high concentrations, such that calcite or vaterite form. The aragonite crystals form via particle assembly, giving nanoparticulate structures analogous to biogenic aragonite, and subsequent growth yields stacked aragonite platelets comparable to structures seen in developing nacre. This mechanism of polymorph selectivity is captured in a theoretical model based on these competing nucleation and growth effects and is completely distinct from the activity of magnesium ions, which generate aragonite by inhibiting calcite. Profiting from these contrasting mechanisms, it is then demonstrated that polyamines and magnesium ions can be combined to give unprecedented control over aragonite formation. These results give insight into calcite/aragonite polymorphism and raise the possibility that organisms may exploit both amine‐rich organic molecules and magnesium ions in controlling calcium carbonate polymorph.

## Graphical abstract

Polyamines, including small molecules and polyelectrolytes, are shown to exert remarkable control over calcite/aragonite polymorphism, where they operate by an entirely different mechanism to magnesium ions and promote aragonite nucleation at exceptionally low concentrations. The aragonite crystals exhibit granular structures analogous to biogenic aragonite and growth via particle assembly yields stacks of aragonite platelets that resemble developing nacre.

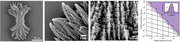

## 1 Introduction

The ability to control polymorph is one of the ultimate challenges in crystallization, where a key goal is to produce specific phases on demand. Some of the best examples of polymorph control are provided by the world of biomineralization, where organisms can select for specific polymorphs with perfect fidelity.^[^ ^1^ ^]^ Significant efforts have therefore been made to identify the strategies by which this is achieved, with a major focus on calcium carbonate. Calcium carbonate is one of the most‐studied model systems for investigating polymorphism, where it crystallizes as three anhydrous polymorphs^[^ ^2^, ^3^ ^]^ of which calcite is the thermodynamically most stable at room temperature, aragonite is only slightly less so, and vaterite is the least stable. All can be precipitated simultaneously from additive‐free aqueous solutions under ambient conditions, where calcite readily forms and vaterite is typically the first phase precipitated at high supersaturations.^[^ ^4^ ^]^ Aragonite invariably only appears as a minor product, in contrast to its high abundance in both biogenic and abiotic environments.

The ability of organisms to achieve selectivity over calcite and aragonite, and in some cases, even switch between the two,^[^ ^1^ ^]^ is typically attributed to specific macromolecules,^[^ ^5^, ^6^ ^]^ or combinations of macromolecules,^[^ ^7^ ^]^ which may select for each polymorph. Magnesium ions also promote the formation of magnesian calcite and ultimately aragonite as the Mg/Ca ratio increases^[^ ^8^, ^9^ ^]^ and may contribute to aragonite formation in vivo.^[^ ^10^ ^]^ However, despite a report of switching between calcite and aragonite using biomacromolecules extracted from aragonite or calcite,^[^ ^5^ ^]^ few have succeeded in generating aragonite using single proteins or protein fragments derived from aragonite^[^ ^11^ ^]^ without combining the macromolecules with more complex environments such as a chitin substrate,^[^ ^12^, ^13^ ^]^ an insoluble *β*‐chitin/silk‐fibroin matrix,^[^ ^6^ ^]^ or the addition of magnesium ions.^[^ ^14^ ^]^

Notably, there are also very few reports of synthetic organic additives that can promote aragonite. Anionic additives are widely used to control the precipitation of calcium carbonate as they are highly effective in controlling the rate of nucleation and the size and shape of the crystals.^[^ ^15^ ^]^ However, this class of molecule only generates calcite or vaterite. The only soluble additives reported to precipitate aragonite are deferoxamine,^[^ ^16^ ^]^ a biogenic iron‐coordinating agent, the custom‐made diblock copolymer poly(sodium 4‐styrene sulfonate‐co‐N‐isopropylacrylamide) (PSS‐co‐PNIPAAM),^[^ ^17^ ^]^ and amphoteric microgel particles.^[^ ^18^ ^]^ Notably, all of these contain basic moieties.

Here, we demonstrate that a range of readily available amine‐rich additives can achieve outstanding selectivity over calcite/aragonite polymorphism, an effect that is further enhanced in the presence of magnesium ions. This runs counter to expectations, where the organic molecules selected to control CaCO_3_ precipitation are principally anionic as are the highly acidic biomacromolecules considered characteristic of calcium carbonate biomineralization. Investigation of the underlying mechanism demonstrates that aragonite nucleation is promoted at extremely low additive concentrations, in contrast to the inhibitory behavior of most soluble additives,but that aragonite growth is then inhibited at high additive concentrations. Coupled with studies of the development of the aragonite crystals, we then present a theoretical model that reproduces this competition between enhanced nucleation and inhibited growth in polymorph selection. Given the increasing recognition that calcium carbonate biomineralization occurs in the presence of a suite of biomolecules,^[^ ^7^, ^19^ ^]^ including many rich in basic residues,^[^ ^13^, ^20^, ^21^, ^22^, ^23^ ^]^ and the close resemblance of the synthetic aragonite crystals to their biogenic counterparts,^[^ ^24^ ^]^ this work contributes to our understanding of the role of basic additives in calcium carbonate biomineralization.

## 2 Results

**Figure 1:**
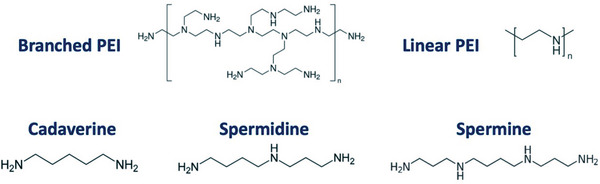
Polyamines used in the study.

Calcium carbonate was precipitated in the presence of amine‐functionalized additives–ranging from branched polyelectrolytes, to linear polyelectrolytes, to small molecules–using the ammonium diffusion method (pH ≈ 9) (**Figure** **1**),^[^ ^25^ ^]^ and a comparison was made with the activity of magnesium ions. Exceptional control was achieved, where most of the molecules generated both calcite and aragonite, and some produced all three polymorphs.

### 2.1 Polymorph Control Using Branched Poly(ethylene imine) (b‐PEI)

**Figure 2:**
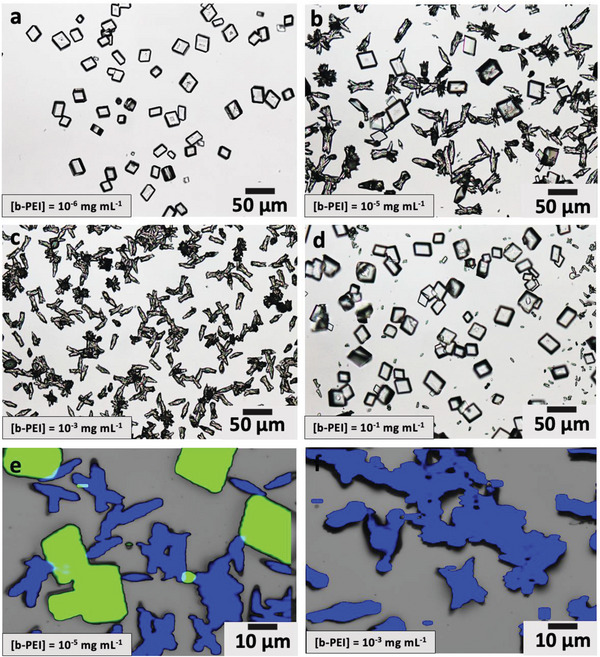
Polymorph control using branched PEI. a–d) Optical images of crystals precipitated at [Ca^2+^] = 10 mm, where pure calcite forms at [b‐PEI] = 10^−6^ mg mL^−1^ (a), while calcite and aragonite form at [b‐PEI] = 10^−5^ mg mL^−1^ (b). c) Pure aragonite is formed at [b‐PEI] = 10^−3^ mg mL^−1^ but d) becomes inhibited at concentrations above this value until pure calcite forms at [b‐PEI] = 10^−1^ mg mL^−1^. e,f) Raman mapping of the CaCO_3_ crystals showing the relationship between polymorph and morphology and showing that calcite (green) and aragonite (blue) forms at [b‐PEI] = 10^−5^ mg mL^−1^ (e) whereas pure aragonite forms at [b‐PEI] = 10^−3^ mg mL^−1^ (f).

Crystallization of CaCO_3_ in the presence of a low molecular weight branched PEI (MW = 1200 g mol^−1^) containing primary, secondary, and tertiary amine groups at a ratio of ≈1:2:1 was studied in detail. This molecule could be used to generate pure phases of calcite and aragonite. Polymorphs were confirmed using optical microscopy and parallel Raman microscopy, where the calcite and aragonite crystals were morphologically distinct (**Figures** **2**e,f). This method is effective in identifying small quantities of a secondary polymorph. Powders were also analyzed using FTIR to determine the polymorphs present (Figure S1, Supporting Information).

While rhombohedral calcite crystals alone formed at [Ca^2+^] = 10 mm and [b‐PEI] = 10^−6^ mg mL^−1^ (Figure 2a), an increase in PEI to only 10^−5^ mg mL^−1^ resulted in the co‐precipitation of aragonite crystals (Figure 2b). The proportion of aragonite then increased with increasing [b‐PEI] until aragonite alone formed at [b‐PEI] ≈ 10^−3^ mg mL^−1^ (Figure 2c). Analysis of the footprint of aragonite crystals precipitated on the glass substrates showed a pseudo‐hexagonal form consistent with nucleation on a (001) face (Figure S2, Supporting Information), where oriented nucleation may have been facilitated by adsorption on the polymer on the substrate.^[^ ^18^ ^]^ TGA showed that <1 wt% of polymer was entrapped within these crystals (Figure S3, Supporting Information). Calcite then started to re‐emerge at higher concentrations of b‐PEI until pure calcite rhombohedra formed at [b‐PEI] = 10^−1^ mg mL^−1^ (Figure 2d). Analysis of calcite crystals grown at [b‐PEI_1,200_] = 2.5 mg mL^−1^ revealed incorporation levels of up to ≈18 wt% (Figure S4, Supporting Information). This is an exceptional level of incorporation and is achieved while maintaining the single crystal character of the calcite host,^[^ ^26^, ^27^, ^28^ ^]^ as demonstrated by single‐crystal XRD and polarized light microscopy (Figure S5, Supporting Information).

Similar trends were also observed when [b‐PEI] was held at 10^−3^ mg mL^−1^ and the calcium concentration was varied. Pure aragonite formed at [Ca^2+^] = 1 mm and increasing proportions of calcite precipitated at [Ca^2+^] = 20 and 50 mm (Figure S6, Supporting Information). However, aragonite remained the dominant phase in all cases. This demonstrates that the ability of polyamines to nucleate aragonite is not restricted to low supersaturations. It is emphasized, however, that polymorph control in the calcium carbonate system is always dependent on supersaturation, and additives (polyamines or magnesium ions) cannot be used to generate aragonite at high supersaturations.

### 2.2 Morphological Control Using b‐PEI

**Figure 3:**
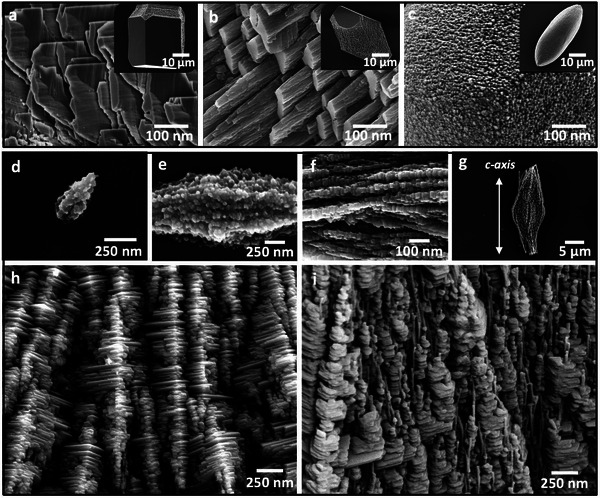
Morphological control using branched PEI. SEM micrographs of calcite crystals precipitated at [Ca^2+^] = 10 mm in the presence of a) [PEI] = 0.25 mg mL^−1^, b) [PEI] = 0.5 mg mL^−1^, and c) [PEI] = 1 mg mL^−1^. d–i) SEM images of aragonite crystals precipitated at [Ca^2+^] = 10 mM and [b‐PEI] = 10^−3^ mg mL^−1^, showing that aragonite formation starts by an assembly of ≈ 20 nm spheroidal nanoparticles that merge together over time (d,e) to form faceted nanocrystals (f). These develop to form stacked layers of hexagonal aragonite platelets co‐aligned along their *c*‐axes (g–i). Co‐alignment of these platelets appears to be guided by granular fibrillar structures that are oriented along the *c*‐axis of aragonite (i).

While the calcite crystals co‐precipitated with the aragonite crystals were rhombohedral in form, higher concentrations of b‐PEI ultimately altered their morphologies, generating crystals that were elongated along the *c*‐axis, which is indicative of preferential binding to the acute steps on the calcite {104} faces (**Figure** **3**a–c).^[^ ^28^, ^29^ ^]^ They also exhibited striking surface features that varied from plate‐like structures at [b‐PEI] = 0.25 mg mL^−1^, to columnar structures at [b‐PEI] = 0.5–1 mg mL^−1^, and ultimately, fine nanogranules at [b‐PEI] > 1 mg mL^−1^ (Figure 3a–c). Cross‐sections through these crystals revealed a solid core and showed that the surface features were confined to the outer ≈500 nm layer (Figure S7, Supporting Information). This is consistent with their formation during the later stages of growth and dissolution/ reprecipitation processes in the crystallization solution.^[^ ^30^, ^31^ ^]^

Scanning electron microscopy (SEM) analysis of the evolution of aragonite crystals precipitated at [Ca^2+^] = 10 mm and [b‐PEI] = 10^−3^ mg mL^−1^ revealed fascinating structures. Elongated crystals ≈ 250 nm in size and that exhibited granular structures were observed within the first 30 min of crystallization (Figure 3d,e) and then developed into spindle‐shaped crystals comprising faceted nanoparticles (Figure 3f) and ultimately crystals with wheatsheaf morphologies (Figure S8, Supporting Information). These subsequently evolved into beautiful structures comprising well‐defined hexagonal aragonite platelets^[^ ^32^, ^33^ ^]^ co‐oriented along their [001] axes (Figure 3g–i). The stacks of platelets appeared to be preceded by fibrillar structures. At this stage of formation, growth along the *c*‐axis was limited by the adjacent crystals in the stacks, while growth within the *ab*‐plane was unconstrained, leading to a platelet morphology (Figure 3h,i). It is intriguing to note that these structures closely resemble the stacks of aligned aragonite nanoplatelets observed during the formation of nacre.^[^ ^24^ ^]^

### 2.3 Exploring Alternative Polyamines

**Figure 4:**
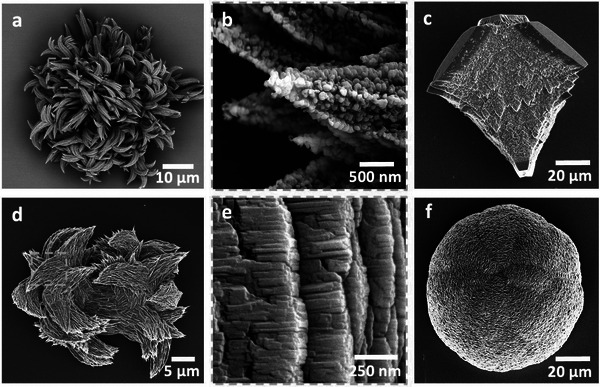
CaCO_3_ crystals precipitated in the presence of linear PEI and cadaverine. a–c) Crystals precipitated at [Ca^2+^] = 10 mm in the presence of linear PEI, MW = 4000 g mol^−1^. Aragonite crystals precipitated at [PEI_4000_] = 10^−3^ mg mL^−1^ display granular textures (a,b). Calcite precipitated at [PEI_4000_] = 1 mg mL^−1^ (c). d–f) Crystals precipitated at [Ca^2+^] = 2.5 mm in the presence of cadaverine. Pure aragonite formed at [cadaverine] = 0.1 mg mL^−1^ (d), where columnar structures formed via fusion of individual platelets at the early stages of aragonite formation are shown (e). Vaterite crystals formed at [cadaverine] = 0.5 mg mL^−1^ (f).

Linear poly(ethylene imine) (PEI) molecules with higher molecular weights (MW = 4000 g mol^−1^ and 10 000 g mol^−1^) showed a similar pattern of behavior to b‐PEI, where calcite rhombohedra formed at very low PEI concentrations, and pure aragonite formed at [Ca^2+^] = 10 mm and [PEI] = 10^−3^ mg mL^−1^ (**Figure** **4**a; Figure S9, Supporting Information). The aragonite crystals displayed a granular texture and comprised 50–150 nm nanoparticles (Figure 4b). Increasing the concentration of PEI to 1 mg mL^−1^ generated calcite with PEI_4,000_ and vaterite with PEI_10,000_ (Figure 4c; Figures S10–S12, Supporting Information). Again, the calcite crystals only exhibited modified morphologies at these high polyelectrolyte concentrations (Figure 4c).

At the opposite end of the size regime, the biogenic amines cadaverine, spermidine, and spermine are small linear hydrocarbons terminated at each end by primary amine groups (Figure 1). Of these, cadaverine enabled selectivity over all three CaCO_3_ polymorphs, where calcite rhombohedra were the sole product at [Ca^2+^] = 2.5 mm and [cadaverine] = 0.05 mg mL^−1^, and an increase in cadaverine to just 0.1 mg mL^−1^ generated pure aragonite (Figure 4d; Figure S13, Supporting Information). The aragonite crystals again exhibited columnar structures comparable to those observed with b‐PEI (Figure 4e). Vaterite precipitated as a pure phase at [cadaverine] > 0.5 mg mL^−1^ (Figure 4f) and converted to calcite after 4 days of incubation in the reaction solution. This polymorph selectivity is extremely unusual, where there are very few reports of additives that can selectively generate all three CaCO_3_ polymorphs.^[^ ^17^, ^34^ ^]^ In contrast, spermidine and spermine only yielded elongated calcite crystals under all conditions explored ([Ca^2+^] = 2.5–20 mm and [biogenic amines] = 0.05–1 mg mL^−1^) (Figures S14 and S15) and were incorporated at up to 7 wt% and 11 wt%, respectively (Figure S16, Supporting Information).

### 2.4 Mechanistic Comparison With Magnesium Ions

**Figure 5:**
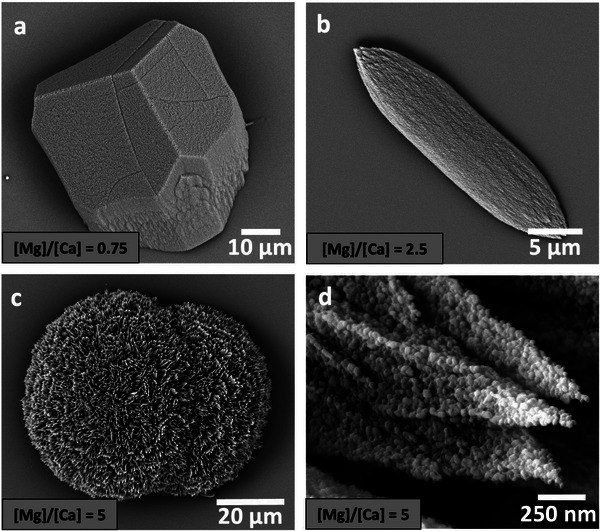
CaCO_3_ crystals precipitated in the presence of Mg^2+^ ions. Optical and SEM images (insets) of crystals precipitated at [Ca^2+^] = 10 mm and a) [Mg^2+^] = 7.5 mm, b) [Mg^2+^] = 25 mm, and c) [Mg^2+^] = 50 mm. Increasing [Mg^2+^] generates calcite crystals that are more elongated along their *c*‐axes when [Mg^2+^]/[Ca^2+^] < 5 (a,b) until aragonite forms at [Mg^2+^]/[Ca^2+^] = 5 (c). d) Aragonite crystal after 10 h showing a granular structure.

It is extremely valuable to compare the activities of the polyamines with those of magnesium ions, where magnesium ions are the go‐to method for generating aragonite at room temperature. Magnesium ions promote aragonite by inhibiting calcite formation, where they substitute for calcium ions in the calcite lattice, thereby increasing the surface energy and solubility.^[^ ^35^, ^36^, ^37^ ^]^ They have little effect on aragonite, which ultimately leads to co‐precipitation of calcite and aragonite, and then aragonite alone at sufficiently high magnesium concentrations. This behavior was confirmed here, where Mg^2+^ ions increasingly modified the morphologies of calcite crystals in the regime [Mg^2+^]/[Ca^2+^] < 5 and [Ca^2+^] = 10 mm (**Figure** **5**a,b; Figure S17, Supporting Information). Increasing amounts of aragonite formed as [Mg^2+^] increased in this regime until pure aragonite formed at [Mg^2+^]/[Ca^2+^] = 5 (Figure 5c). Aragonite formed in the presence of Mg^2+^ ions also exhibited granular structures (Figure 5d). Much longer induction times were recorded for aragonite formation in the presence of magnesium ions (4 h) as compared with the polyamines (30 min) at identical calcium concentrations, as estimated using optical microscopy.

In contrast, aragonite co‐precipitates with calcite crystals with perfect rhombohedral morphologies at low concentrations of b‐PEI (MW = 1200 g mol^−1^) (Figure 2b). No change in the calcite morphology is observed as calcite is replaced by aragonite with increasing b‐PEI, and the co‐existent calcite and aragonite crystals are of comparable sizes (Figure 2b; Figure S2, Supporting Information). This shows that the additive has a strong effect on aragonite at these concentrations but has no effect on calcite. Much higher concentrations of polyamines (≥0.25 mg mL^−1^) are required to induce habit changes of calcite (Figure 3a–c),^[^ ^38^ ^]^ while the cationic polyelectrolytes modify the morphologies and decrease the sizes of the aragonite crystals at relatively low concentrations (Figure 2d). The mechanism by which the polyamines generate aragonite is therefore distinct from that occurring with magnesium ions, where they promote aragonite rather than inhibiting calcite.

### 2.5 Rationalizing Polymorph Control

The dependence of calcite/ aragonite formation on the concentration of polyamines can be rationalized by constructing a toy model that captures the competition between enhanced nucleation and inhibited growth in polymorph control (see also Section 4; Figure S18, Supporting Information). Taking the case of b‐PEI, this additive 1) induces aragonite but has a negligible effect on calcite at low concentrations, and 2) induces aragonite formation at exceptionally low additive concentrations (0.01 *µ*g mL^−1^), which precipitates alongside calcite until [b‐PEI] ≈ 1 *µ*g mL^−1^ where aragonite is the sole phase formed. 3) Calcite then re‐emerges as [b‐PEI] is further increased until all aragonite is inhibited. This parallel action of enhanced nucleation and inhibited growth is consistent with the principle that additives that are good nucleators are also good binders (and therefore inhibitors).^[^ ^39^ ^]^

In our model, the aragonite crystals are assumed to form via the diffusion‐limited assembly of primary nanoparticles which nucleate in association with the polyamines. The nucleation rate of the aragonite assemblies will therefore be directly proportional to the polyamine concentration *c*. The rate of aragonite growth, on the other hand, is assumed to be inhibited by the additive to a degree that is proportional to the additive coverage on the aragonite surfaces, where the coverage depends on *c* according to a Langmuir adsorption isotherm. The precipitation of calcite in the model is unaffected by the additive because experimentally, the additive does not have a discernible effect on the growth of calcite over the *c* range of interest and the free parameters of the model are chosen to qualitatively reproduce the observations for b‐PEI.

**Figure 6:**
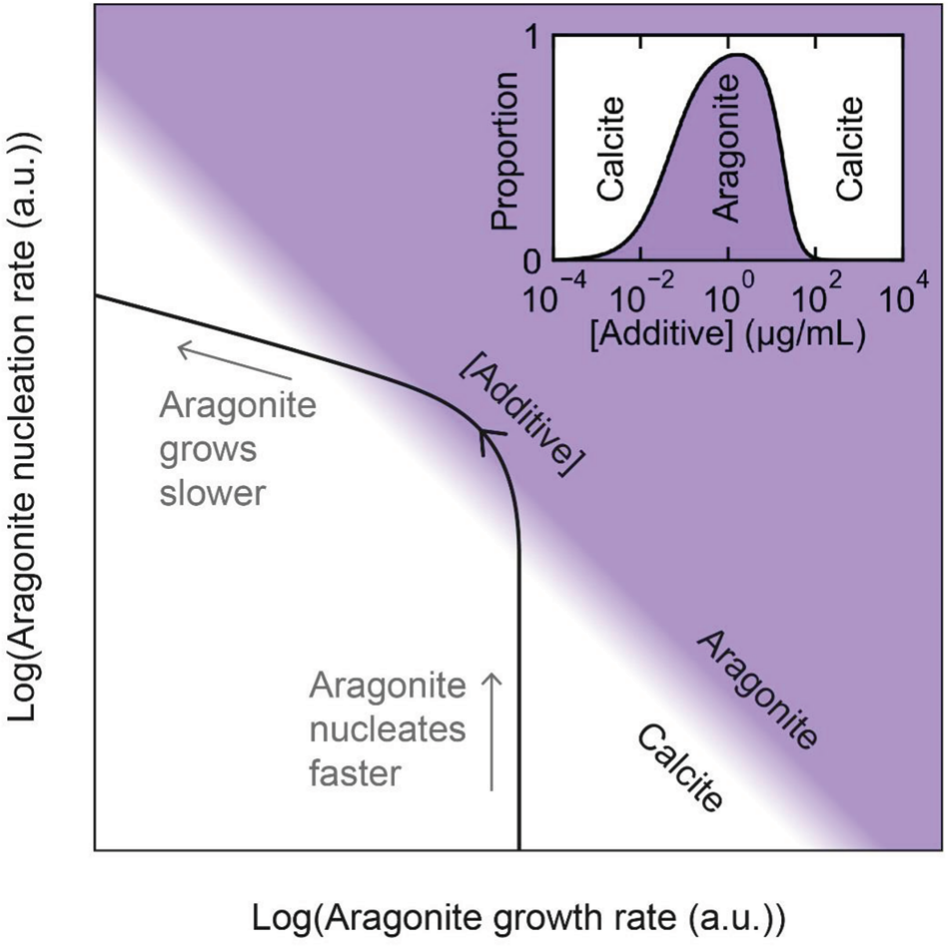
Theoretical toy model capturing the competition between calcite and aragonite in the presence of a polyamine additive. The purple and white colors represent a high abundance of aragonite and calcite, respectively. The contour shows how the rates of nucleation and growth vary for aragonite as a function of (additive), where the arrow shows the direction of increasing (additive). The accelerated nucleation causes aragonite to dominate but the subsequent growth inhibition suppresses aragonite back out of existence. The inset shows the relative proportion of the two polymorphs moving along the contour.

The model reveals how the relative proportion (volume fraction) of aragonite depends on the relative rates of nucleation and growth of the two phases (**Figure** **6**). In particular, due to its functional dependence on the additive concentration, the model predicts two distinct stages consistent with the experimental observations: an initial increase in the abundance of aragonite due to accelerated nucleation, followed by a decreased abundance as the enhanced surface coverage smothers the aragonite phase back out of existence.

### 2.6 Combining Mg^2+^ Ions and b‐PEI

**Figure 7:**
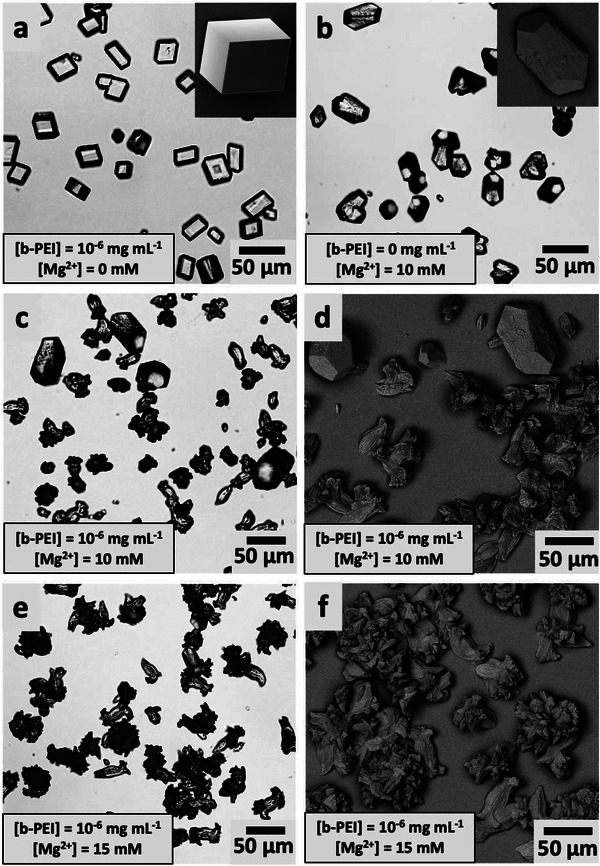
a,c,e) Optical microscopy and b,d,f) SEM images of crystals precipitated at [Ca^2+^] = 10 mm in the presence of b‐PEI and Mg^2+^ ions. [b‐PEI] = 10^−6^ mg mL^−1^ (a), [Mg^2+^] = 10 mm (b), [b‐PEI] = 10^−6^ mg mL^−1^ (c,d), and [Mg^2+^] = 10 mm (e,f) [b‐PEI] = 10^−6^ mg mL^−1^ and [Mg^2+^] = 15 mm.

Given that Mg^2+^ ions and the polyamines promote aragonite via different mechanisms, it may be expected that in combination they could offer a very effective route to generating aragonite at low additive concentrations. This was explored by precipitating calcium carbonate from solutions containing both Mg^2+^ and b‐PEI (**Figure** **7**). Calcite was the sole polymorph formed in solutions comprising i) [Ca^2+^] = 10 mm and [b‐PEI] = 10^−6^ mg mL^−1^ and ii) [Ca^2+^] = [Mg^2+^] = 10 mm (Figure 7a,b). In contrast, both calcite and aragonite crystals formed in solutions containing [Ca^2+^] = 10 mm, [b‐PEI] = 10^−6^ mg mL^−1^ and [Mg^2+^] = 10 mm (Figure 7c,d). A slight increase of [Mg^2+^] = 15 mm, while keeping constant [Ca^2+^] = 10 mm and [b‐PEI] = 10^−6^ mg mL^−1^, yielded pure aragonite (Figure 7e,f). This can be compared with the [b‐PEI] = 10^−3^ mg mL^−1^ or the [Mg^2+^] = 50 mm required to generate pure aragonite when these additives are present on their own. These results therefore show that due to the different mechanisms by which they generate aragonite, Mg^2+^ ions and polyamine additives can act in combination to generate aragonite at much lower concentrations than either would do individually.

### 2.7 Investigating the Mechanism of Aragonite Growth

**Figure 8:**
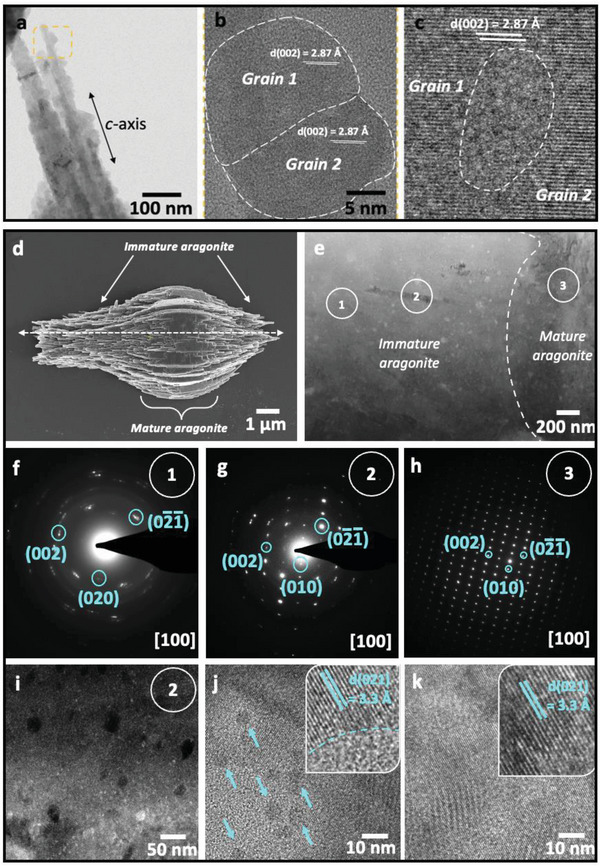
TEM analysis of early stages of formation of aragonite crystals. Crystals were precipitated at [Ca^2+^] = 10 mm and [b‐PEI] = 10^−3^ mg mL^−1^. a) Fibers with granular structures that are elongated along the *c*‐axis, b,c) HRTEM images of the aragonite nanoparticles, where some domains that are devoid of lattice fringes (dashed line) and are consistent with ACC are observed at grain boundaries (c). d) Aragonite crystal terminated by needle‐like structures and e) STEM image of a thin lamella prepared parallel to the *c‐*axis of the crystal shown in (d). f–h) SAED patterns of lamella taken from the areas indicated in (e), showing angular spread decreasing from 15° to 5° (from areas 1 to 2), indicating a gradual co‐alignment of the nanograins, until the mature aragonite diffracts as a perfect single crystal (area 3). i) HAADF–STEM image of the immature region of the aragonite lamella. j,k) Electron beam irradiation of the amorphous particles results in the formation of ≈5 nm aragonite nanograins (arrows in (j)) that grow on prolonged electron irradiation to form larger continuous domains of aragonite (k).

Finally, we exploited this system to investigate the mechanism of growth of aragonite crystals in the presence of soluble organic additives. Focusing on b‐PEI, transmission electron microscopy (TEM) analysis demonstrated that the initial aragonite crystals formed via the assembly of ≈20 nm nanoparticles, where these particles were co‐oriented along their *c*‐axes (**Figure** **8**a), as confirmed by the continuity of lattice fringes in HRTEM images (Figure 8b). Closer examination of the boundary between adjacent aragonite nanoparticles revealed non‐crystalline domains (Figure 8c) consistent with amorphous calcium carbonate (ACC). Given the supersaturations employed in these experiments, some ACC can be expected to form as a precursor phase.^[^ ^40^, ^41^ ^]^ The presence of ACC at junctions between coherent aragonite^[^ ^42^, ^43^, ^44^ ^]^ and vaterite^[^ ^45^ ^]^ nanoparticles in crystalline superstructures has previously been associated with assembly‐based crystallization within an ACC phase.

Scanning TEM (STEM) was also used to probe the internal structure of a developing aragonite crystal, where a thin lamella was cut parallel to the *c*‐axis (Figure 8d,e). Figure 8e shows two distinct regions corresponding to newly‐formed (immature) aragonite needles and more mature aragonite platelets (that diffract as single crystals). SAED analyses show that the angular spreads gradually decreased from 15° to 5° during the transition from the immature aragonite to more mature material until it ultimately diffracted as a perfect single crystal (Figure 8f–h) and continuous lattice fringes were observed (Figure S19, Supporting Information). Imaging of the immature aragonite with bright‐field STEM and HAADF revealed the presence of 20–75 nm particles that were devoid of lattice fringes and appeared bright in bright‐field STEM images and dark in the HAADF images (Figure 8e,i; Figure S20, Supporting Information). This is consistent with the lower density of ACC than aragonite (1.6 g cm^−3^ vs 2.9 g cm^−3^).^[^ ^46^ ^]^ Final confirmation of their identity as ACC was obtained by irradiation with the electron beam which led to the formation of ≈5 nm crystalline nanoparticles that grew on prolonged electron irradiation to form larger aragonite nanodomains (Figure 8j,k).

## 3 Discussion

It is difficult to generate aragonite in aqueous solutions under ambient conditions in the absence of magnesium ions. This is reflected in the challenge of identifying universal factors (with the exception of magnesium ions and elevated temperatures) that drive aragonite formation.^[^ ^47^ ^]^ For example, high aragonite fractions have been reported around room temperature under additive‐free conditions in a narrow range of supersaturations,^[^ ^48^ ^]^ at specific positions in a gel column in a double‐diffusion reaction,^[^ ^49^ ^]^ within cylindrical nanopores,^[^ ^50^ ^]^ in the vicinity of CO_2_/NH_3_ microbubbles at pH 9.7–10.5,^[^ ^51^ ^]^ and in a constant composition environment at an optimal pH of 11.^[^ ^52^ ^]^ Consideration of aragonite formation in the environment has additionally highlighted the importance of high CO_2_ degassing rates, and thus the supply of carbonate ions.^[^ ^47^, ^53^ ^]^

Ostwald's rule describes the observation that many polymorphic compounds crystallize via a sequence of increasingly stable phases,^[^ ^54^ ^]^ and is presumed to occur when the energy barriers associated with the formation of each metastable phase are lower than that leading directly to the most stable phase. It is rarely followed in the case of calcium carbonate, however, where aragonite does not form as a precursor to calcite under ambient conditions. This demonstrates that although calcite and aragonite are very similar in thermodynamic stability, the energy barrier to aragonite nucleation is greater than to calcite in the absence of additives. Aragonite therefore only forms under reaction conditions that either actively promote its nucleation or which inhibit the formation of calcite (as occurs with magnesium ions).

Our data show that amine‐rich molecules promote aragonite formation under conditions where they have little effect on calcite. This is consistent with them acting as effective binders to aragonite nuclei, where additives that are good nucleators are also good binders (and therefore, inhibitors).^[^ ^39^ ^]^ Given the dependence of nucleation rates on interfacial energies predicted by classical nucleation theory, this suggests that the interfacial energies of aragonite nuclei experience a greater decrease on binding of amines than do those of calcite. While experiments and modeling studies have shown that amine‐functionalize molecules and amino acids can bind to the steps parallel to the {104} faces of calcite,^[^ ^27^, ^55^ ^]^ the equivalent data is not available for aragonite. However, the potential expression of multiple crystal faces on aragonite and the absence of well‐defined cleavage planes is consistent with the interfacial energies of aragonite being sensitive to additive binding.

It is also interesting to compare the activities of the different molecules used in this study. As it is challenging to provide a measure of concentration that allows molecules with different sizes and structures to be compared, molecular concentrations with different units, the concentration with respect to the amine groups present, and the polymorphs generated are summarized in Table S1, Supporting Information. b‐PEI and linear PEI show similar activities, where both generate aragonite at 10^−3^ mg mL^−1^ and similar concentrations of amine functional groups (1.5 × 10^−5^ and 2.3 × 10^−5^ m, respectively). Notably, linear PEI only contains secondary amines, while b‐PEI contains primary and secondary amines at a ratio of ≈2:1. This demonstrates that the reduced complexing ability of secondary as compared with primary amines has little impact on their ability to promote aragonite.

Cadaverine, in contrast, shows a different behavior to spermidine and spermine, where it can generate pure phases of all three polymorphs as opposed to just calcite with spermidine and spermine. Notably, all are linear (Figure 1), all are terminated by primary amine groups, and all are employed at comparable concentrations of functional groups (Table S1, Supporting Information). However, spermine and spermidine also contain secondary amines in addition to the terminal primary amines. No trend is observed with the charge on the molecules, where all are cationic under the experimental conditions: spermine (pK_a_1 = 10.1, pK_a_2 = 8.9, and pK_a_3 = 8.1), spermidine (pK_a_1 = 10.81, pK_a_2 = 9.94, and pK_a_3 = 8.40), and cadaverine (pK_a_1 = 10.25 and pK_a_2 = 9.13). Cadaverine is the shortest of the molecules, which may enable the two amine groups to bind cooperatively to an emerging nucleus or crystal surface.^[^ ^56^ ^]^ The longer spermidine and spermine are expected to have a greater conformational freedom in solution, such that there is a greater loss of entropy on binding to the crystal surface. Simulations have also provided insight into the influence of spermine and spermidine on calcite growth.^[^ ^27^ ^]^ The secondary amine group present in both biogenic amines interacts strongly with the calcite terrace, but does not bind to step or kink sites. It is likely that the secondary amines further stabilize bound states of spermine and spermidine at growth sites by interacting with the terraces.

The early stages of growth occur via the oriented assembly of aragonite nanoparticles, where assembly‐based growth has been observed for many poorly‐soluble compounds such as TiO_2_,^[^ ^57^ ^]^ CdSe, haematite (*α*‐Fe_2_O_3_),^[^ ^58^ ^]^ and magnetite (Fe_3_O_4_)^[^ ^59^, ^60^ ^]^ and is driven by short‐range attractive forces; the crystalline nanoparticles undergo translations and rotations to explore low‐energy configurations in a process that can be modified using soluble additives.^[^ ^61^ ^]^ It is emphasized, however, that new nanoparticles only appear to form near the surface of the aragonite crystals and no burst of nanoparticle nucleation is observed in bulk solution. This localized nucleation could be due to interfacial gradients, as reported recently for the formation of haematite crystals in the presence of oxalate,^[^ ^62^ ^]^ or perhaps due to surface‐bound additives providing a site for heterogeneous nucleation. Subsequent growth then occurs under control of the additives, where the constituent nanoparticles grow preferentially in the less constrained *ab*‐plane to give stacks of platelets.

Such a mechanism also rationalizes how b‐PEI and linear PEI can promote aragonite at such low concentrations. Pure aragonite is formed at 8.33 × 10^−7^ m (b‐PEI) and 2.5 × 10^−7^ m (linear PEI_4000_) in 10^−2^ m Ca^2+^ solutions, where the equivalent concentrations of amine groups are 1.51 × 10^−5^ m (b‐PEI) and 2.27 × 10^−5^ m (linear PEI_4000_). If a nucleation burst were to generate 2–3 nm particles that then assembled to form the aragonite crystals, a back‐of‐the‐envelope calculation estimates ≈100 nuclei per polymer molecule. In contrast, the formation of an initial population of nuclei that then grow into ≈20 µm aragonite crystals places the polymer at a huge excess.

It is also valuable to compare the structures of the synthetic aragonite formed here in the presence of polyamines with those of aragonite biominerals. TEM analysis of nacre has shown that it possesses a similar nanoparticulate ultrastructure,^[^ ^44^ ^]^ as does aragonite precipitated on an insoluble protein matrix^[^ ^42^ ^]^ and aragonite formed at elevated temperatures in the absence of additives.^[^ ^63^ ^]^ Such a particle aggregation mechanism is also consistent with the morphologies of aragonite particles grown in the presence of additives, where these exhibit branching from a single crystal core to give characteristic wheatsheaf^[^ ^64^ ^]^ or spherulitic forms^[^ ^65^ ^]^ that are indicative of imperfect nanoparticle alignment. Notably, aragonite spherulites have been observed in many species of coral,^[^ ^66^ ^]^ where randomly‐oriented aragonite nanoparticles were observed in an ACC matrix, close to the growth front of existing spherulitic aragonite. Subsequent competitive growth then delivered a spherulitic structure. At a larger length‐scale, characterization of the prism/nacre interface revealed fiber‐like arrays of stacked aragonite nanocrystals similar to those shown in Figure 3e,f.^[^ ^24^ ^]^ This demonstrates that far from these structural features being a signature of biological control, they appear to be a consequence of the general growth mechanism of aragonite and can be tuned using soluble additives.

## 4 Conclusion

In summary, this work demonstrates that polyamines can exert exceptional control over calcium carbonate polymorph, where all three anhydrous phases can be selected by tuning the reaction conditions. This behavior highlights the failure of anionic molecules–synthetic or biological in origin–to generate aragonite in bulk solution, although, aragonite has been observed when certain acidic proteins are adsorbed on specific substrates such as chitin.^[^ ^67^ ^]^ While most additives inhibit nucleation, polyamines promote aragonite formation, an effect that is achieved at exceptionally low concentrations. Their activity is therefore entirely distinct from magnesium ions, which play key roles in the formation of calcium carbonate in the environment^[^ ^3^, ^8^ ^]^ and whose ability to generate aragonite by inhibiting calcite is well‐documented.^[^ ^35^ ^]^ Following traditional studies of individual biomineralization proteins, high throughput methods have now revealed that large families of proteins are associated with calcite and aragonite in mollusk shells.^[^ ^7^, ^19^ ^]^ Although these families are typically quite distinct from each other, no overarching patterns linking structure to polymorph have been identified, and it is likely that molecules act in combination to control crystallization. Our demonstration that magnesium ions and polyamines can act together to generate aragonite at much lower concentrations than either could do individually further complicates this picture. Simple additive systems such as the one described here therefore provide a unique opportunity to study calcium carbonate polymorphism using experimental and modeling methods, giving insight into biological control strategies and the function of proteins.

## 5 Experimental Section

### Materials

CaCl_2_·2H_2_O, MgCl_2_·6H_2_O, (NH_4_)_2_CO_3_, biogenic amines (cadaverine, spermidine, and spermine), and cationic polyelectrolytes (linear and branched poly(ethyleneimine) (PEI) (MWs = 600–25 000 g mol^−1^)) were purchased from Sigma–Aldrich and were used as received. All solutions were prepared using Milli‐Q deionized (DI) water. Glass slide substrates were thoroughly cleaned by soaking in Piranha solution (H_2_SO_4_ : H_2_O_2_ – 70 vol% : 30 vol%), washed with DI water followed by ethanol, and dried using N_2_(g) stream, prior to use.

### CaCO_3_ Mineralization in the Presence of Positively Charged Additives

Calcium carbonate (CaCO_3_) was precipitated using the ammonium carbonate diffusion method^[^ ^25^ ^]^ in the presence of the cationic additives (biogenic amines or cationic polyelectrolytes). The glass substrates were deposited at the bottom of multi‐well plates containing [Ca^2+^] = 1–50 mm and desired amounts of cationic additives (0–2.5 mg mL^−1^) or magnesium ions (2.5–50 mm). Mineralization was carried out by placing the reaction mixture inside a sealed desiccator containing 2 g of (NH_4_)_2_CO_3_ placed in a Petri‐dish covered with Parafilm punctured several times with a needle. After reaction completion, the substrates supporting the crystals were washed several times with DI water and then ethanol, followed by gentle drying using N_2_(g) stream, prior to characterization.

### Electron Microscopy

The CaCO_3_ crystals were imaged with scanning electron microscopy (SEM) using a FEI NanoSEM Nova 450. The samples were mounted on SEM stubs using carbon adhesive discs and coated with a 4 nm iridium layer, prior to imaging. Cross‐sections through the PEI/calcite crystals were prepared using focused ion beam (FIB) milling with a FEI Helio G4 CX dual beam‐high resolution monochromated FEG SEM instrument. A selected area of the crystal was pre‐coated with 2 µm thick Pt. The operating voltage was 30 kV and the beam currents were varied between 0.1 and 5 nA.

Transmission electron microscopy (TEM) analyses of the vaterite and aragonite crystals were carried out using a FEI Titan3 Themis G2 S/TEM operated at 300 kV with a FEI Super‐X energy dispersive X‐ray (EDX) system and a Gatan OneView CCD camera. The TEM images were collected at a screen current of 3 nA and the STEM imaging and mapping were carried out using a probe current of 30 pA. The analysis of the internal structure of the aragonite crystals was carried out by preparing a thin lamella using FIB‐SEM, which was transferred to a copper TEM grid using a Kleindiek micromanipulator. High‐angle annular dark‐field scanning TEM (HAADF‐STEM) imaging in conjunction with EDX analysis mapping was carried out to unravel the composition and structure of aragonite crystals grown in the presence of cationic polyelectrolytes.

### Thermogravimetric Analysis (TGA)

Thermogravimetric analyses were performed from 20 °C to 850 °C in air, using a TA‐Instruments Q600 operating at 10 °C min^−1^. The samples were bleached prior to characterization to remove the surface bound organic matter.^[^ ^68^ ^]^ Calcination of the pure calcite crystals showed an onset of decomposition at 650 °C, giving a weight loss of 44.0 wt% that is ascribed to the release of CO_2_(g), leaving a residue of 56.0 wt% that corresponds to CaO(s).

Pyrolysis of the biogenic amines/calcite composite single crystals indicated that 7 wt% of spermidine and 11 wt% of spermine were incorporated within calcite. TGA analyses of the PEI /calcite crystals showed a weight loss of 16 wt% below 650 °C due to the thermal decomposition of PEI incorporated within calcite. Assuming that all calcite crystals decompose at 800 °C into CO_2_(g) + CO(s), then an excess of ≈1.9 wt% of PEI remains in the crucible alongside the CO(s) residues. This corresponds to the remaining PEI that does not fully decompose on annealing. Overall, this equates to ≈18 wt% of PEI incorporated within calcite single crystals.

TGA analysis of aragonite grown in the presence of b‐PEI_1,200_ showed that less than 1 wt% of polymer was occluded within the intracrystalline aragonite structure.

### Single‐Crystal XRD

Suitable spermine/calcite and PEI/calcite composite crystals were fixed to micro‐loops using an oil and mounted on a Rigaku XtaLAB Synergy Custom X‐ray diffractometer (Cu‐K*α* radiation 𝜆 = 1.54184 Å) and diffraction data were collected on a HyPix‐6000HE hybrid photon counting (HPC) detector. The crystals were kept at 293 K during data collection, which were carried out for a 2*θ* range = 23.064–134.602°. Initial data collection, indexing, and integration procedures were performed using the Rigaku Oxford Diffraction software; CrysAlisPro. The resulting data were solved and refined using Olex2,^[^ ^69^ ^]^ with the ShelXT^[^ ^70^ ^]^ structure solution program using Intrinsic Phasing and refined with the ShelXL^[^ ^71^ ^]^ refinement package using Least Squares minimization.

### Other Measurements

Optical micrographs of the CaCO_3_ crystals were recorded using a Nikon Eclipse LV100 polarizing microscope, equipped with both transmitted and reflected light sources. Fourier‐transform infrared (FTIR) spectra were acquired over the mid‐infrared region (600–2000 cm^−1^) using a Perkin–Elmer ATR‐IR instrument. Individual crystal polymorphs were obtained by Raman spectroscopy, using a Renishaw 2000 Raman Microscope equipped with a 785 nm diode laser. Raman mapping of the CaCO_3_ crystals was carried out using a Horiba HR Evolution Confocal Raman Microscope 50 W laser equipped with a 534 nm laser and an edge filter allowing acquisition down to 50 cm^−1^. An 1800 grooves mm^−1^ grating and a confocal hole open at 100 µm providing a spectral resolution of <0.2 cm^−1^ and a spatial resolution of <0.5 µm (lateral) and <1.5 µm (depth). Mapping was carried out using a 51 × 153 point in a 115 × 156 µm area and 10 s dwell time, and polymorph identification between calcite and aragonite was carried out by selecting the characteristic peaks at 281 cm^−1^ for calcite and 208 cm^−1^ for aragonite.

## Conflict of Interest

The authors declare no conflict of interest.

## Supporting information

Supporting Information

## Data Availability

The data that support the findings of this study are openly available in Research Data Leeds Repository at https://doi.org/10.5518/1177, reference number 72.
