# Supplementary material for: Polyamines Promote Aragonite Nucleation and Generate Biomimetic Structures
Source: Adv Sci (Weinh). 2022 Nov 20;10(1):2203759. doi: 10.1002/advs.202203759 (PMC9811428; doi:10.1002/advs.202203759)
Supplement: Supplementary file 1 — Supporting Information [file ADVS-10-2203759-s001.pdf]

SUPPORTING INFORMATION

Polyamines Promote Aragonite Nucleation and Generate Biomimetic Structures

Ouassef Nahi,[1] Alexander N. Kulak,[1] Shuheng Zhang,[1] Xuefeng He,[1] Zabeada Aslam,[2] Martha A. Ilett,[2] Ian J. Ford,[3] Robert Darkins[3] and Fiona C. Meldrum[1*]*

[1] School of Chemistry, University of Leeds, Woodhouse Lane, Leeds, LS2 9JT, UK

[2] School of Chemical and Process Engineering, University of Leeds, Woodhouse Lane, Leeds, LS2 9JT, UK

[3] London Centre for Nanotechnology, University College London, 17-19 Gordon Street, London, WC1H 0AH, UK

1. Toy Model of Polymorph Selection

Aragonite crystals form *via* an assembly-based mechanism of primary nanoparticles (NPs). For simplicity, we omit any transient precursor phases. If the additives provide a substrate for NP nucleation, then the NPs will nucleate at a rate ($\alpha_0 c$) that is proportional to the a constant α_0 and the additive concentration c. We further suppose that NP aggregation is irreversible, mediated by monomer addition, and that clusters exceeding size s are removed from solution (a mathematical trick for achieving steady state). In steady state, the rate of i-mer nucleation will be independent of i,

$$J_A = \alpha_1 n_1^2 = \cdots = \alpha_{i-1} n_{i-1} n_1 = \cdots$$

for constants α_i, where n_i is the density of i-mers subject to $n_i = 0$ for $i > s$. Also, at steady state, the rate of NP creation must balance with the rate of NP removal (*via* aggregation), *i.e.*

$$\alpha_0 c = \sum_{i=1}^{s} \alpha_i n_i n_1 = s J_A$$

It follows that the nucleation rate of the aragonite assemblies J_A will be proportional to the additive concentration *c*,

$$J_A = jc$$

where *j* is a proportionality constant (equal to α_0/s).

Let R_A be the normal growth rate of the aragonite assemblies. We make no assumption about the growth mechanism except that it is inhibited according to $R_A = k\,(1 - \theta)$ for a constant *k*, where the additive coverage θ on the aragonite surface is given by a Langmuir adsorption isotherm with an equilibrium constant *K*,

$$\theta = \frac{Kc}{Kc + 1}$$

We suppose calcite has a fixed nucleation rate J_C and growth rate R_C that are both unaffected by the additive.

To model the birth and growth process, suppose that each phase *i* (where *i* = C or A) nucleates at a rate J_i and grows at a rate R_i, where both are constant through time. Approximating each crystal as spherical, the number density of crystals of phase *i* and radius *r* at time *t* will be:

$$n_i(r,t) = J_i H\left(t - \frac{r}{R_i}\right)$$

where *H* is the Heaviside step function. Of all precipitated crystals, the total volume fraction composed of aragonite will then be:

$$P_A(t) = \frac{\int_0^{r_{max}} n_A(r,t) r^3 dr}{\int_0^{r_{max}} [n_A(r,t) + n_C(r,t)] r^3 dr}$$

where the case $P_A = 0$ denotes pure calcite and $P_A = 1$ denotes pure aragonite. The cluster sizes are capped at r_{max} due to the process of large-cluster removal. However, we can make r_{max} arbitrarily large such that it becomes irrelevant,

$$P_A = \left(1 + \frac{J_C R_C^4}{J_A R_A^4}\right)^{-1}$$

This equation can be rewritten:

$$P_A = (1 + \xi c^{-1}(1 + Kc)^4)^{-1}$$

in terms of two parameters: the equilibrium constant K, and:

$$\xi = \frac{J_C R_C^4}{j k^4}$$

Based on a qualitative description of the experimental results (specifically, the appearance of aragonite at $c \approx 0.01$ μg mL^{-1} and its peak dominance at $c \approx 1$ μg mL^{-1}) we estimate:

$$\xi \approx 0.05\, \mu g\ mL^{-1}$$
$$K \approx 0.2$$

where K has a reference state of $c = 1$ μg mL^{-1}.

The model yields a family of solutions that correspond to the above values of ξ and K and arbitrary values of k/R_C (experimentally undetermined). See Figure S18 which is simplified in Figure 6. The inset is a plot of P_A against c. The reason the model never quite reaches $P_A = 1$

(pure aragonite) is because we assumed calcite to be unaffected by the additive, when really it should be (indirectly) inhibited due to its competition with the aragonite NPs for solutes.

SUPPORTING INFORMATION

Table S1: Comparison of measures of concentrations of the amine-functionalized additives used, the concentration of amine groups present, and the polymorphs produced

**b-PEI MW = 1,200 g mol^{-1}**	**10^{-6} mg mL^{-1}**	**10^{-3} mg mL^{-1}**	**10^{-1} mg mL^{-1}**	**1 mg mL^{-1}**
	8.33×10^{-10} M	8.33×10^{-7} M	8.33×10^{-5} M	8.33×10^{-4} M
**primary amine**	9.16×10^{-9} M	9.16×10^{-6} M	9.16×10^{-4} M	9.16×10^{-3} M
**primary + secondary amine**	1.51×10^{-8} M	1.51×10^{-5} M	1.51×10^{-3} M	1.51×10^{-2} M
**polymorph**	**calcite**	**aragonite**	**calcite**	**calcite**
**Linear PEI 4,000 g mol^{-1}**	**10^{-6} mg mL^{-1}**	**10^{-3} mg mL^{-1}**	**10^{-1} mg mL^{-1}**	**1 mg mL^{-1}**
	2.5×10^{-10} M	2.5×10^{-7} M	2.5×10^{-5} M	2.5×10^{-4} M
**secondary amine**	2.27×10^{-8} M	2.27×10^{-5} M	2.27×10^{-3} M	2.27×10^{-2} M
**polymorph**	**calcite**	**aragonite**	**calcite**	**calcite**
**Linear PEI 10,000 g mol^{-1}**	**10^{-6} mg mL^{-1}**	**10^{-3} mg mL^{-1}**	**10^{-1} mg mL^{-1}**	**1 mg mL^{-1}**
	1×10^{-10} M	1×10^{-7} M	1×10^{-5} M	1×10^{-4} M
**secondary amine**	2.27×10^{-8} M	2.27×10^{-5} M	2.27×10^{-3} M	2.27×10^{-2} M
**polymorph**	**calcite**	**aragonite**	**vaterite**	**vaterite**
**Cadaverine MW = 102.18 g mol^{-1}**		**5×10^{-2} mg mL^{-1}**	**10^{-1} mg mL^{-1}**	**1 mg mL^{-1}**
		4.89×10^{-4} M	9.79×10^{-4} M	9.79×10^{-3} M
**primary amine**		9.78×10^{-4} M	1.96×10^{-3} M	1.96×10^{-2} M
**polymorph**		**calcite**	**aragonite**	**vaterite**
**Spermidine MW = 145.25 g mol^{-1}**		**5×10^{-2} mg mL^{-1}**	**10^{-1} mg mL^{-1}**	**1 mg mL^{-1}**
		3.44×10^{-4} M	6.88×10^{-4} M	6.88×10^{-3} M
**primary amine**		6.88×10^{-4} M	1.38×10^{-3} M	1.38×10^{-2} M
**primary + secondary amine**		1.03×10^{-3} M	2.06×10^{-3} M	2.06×10^{-2} M
**polymorph**		**calcite**	**calcite**	**calcite**
**Spermine MW = 202.35 g mol^{-1}**		**5×10^{-2} mg mL^{-1}**	**10^{-1} mg mL^{-1}**	**1 mg mL^{-1}**
		2.47×10^{-4} M	4.94×10^{-4} M	4.94×10^{-3} M
**primary amine**		4.94×10^{-4} M	9.88×10^{-4} M	9.88×10^{-3} M
**primary + secondary amine**		9.88×10^{-4} M	1.98×10^{-3} M	1.98×10^{-2} M
**polymorph**		**calcite**	**calcite**	**calcite**
**Mg^{2+}**		**7.5×10^{-3} M**	**2.5×10^{-2} M**	**5.0×10^{-2} M**
**polymorph**		**calcite**	**calcite**	**aragonite**

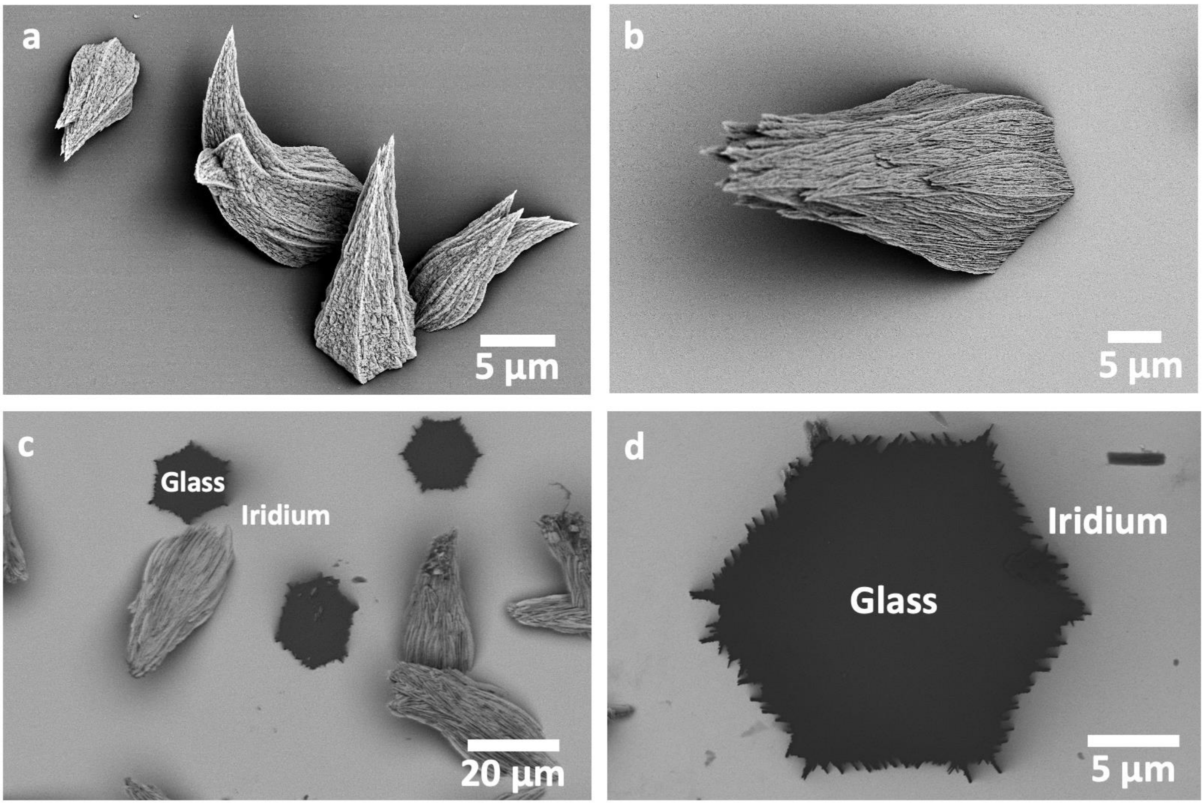

Figure S1. (a-d) SEM micrographs of a pure phase of aragonite crystals precipitated at $[Ca^{2+}]$ = 10 mM and [b-PEI] = 10^{-3} mg mL^{-1}. (c-d) Imprints of iridium coated aragonite crystals nucleated with a pseudo-hexagonal shape on the glass substrate, after removal of the crystals. This suggests that aragonite crystals could be heterogeneously nucleated on the glass substrate with a pseudo-[001] orientation.

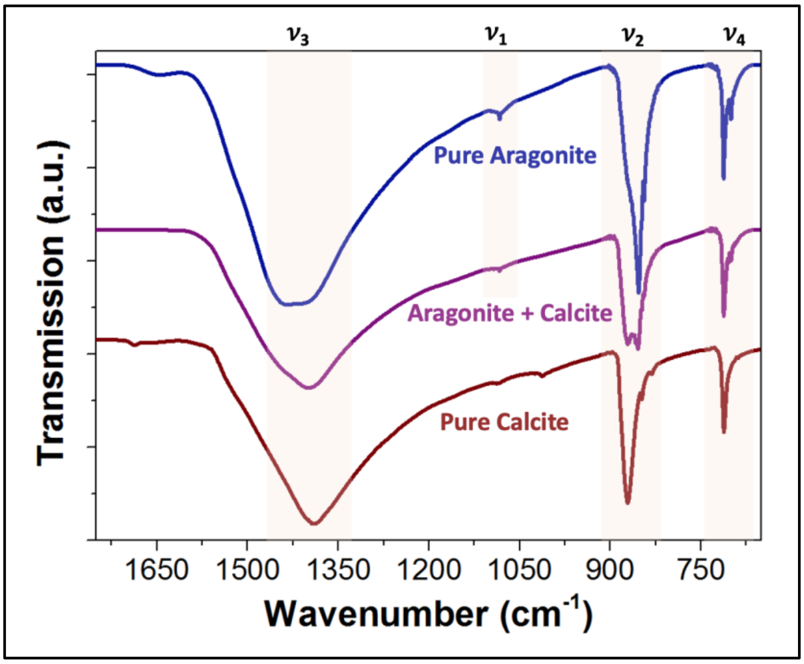

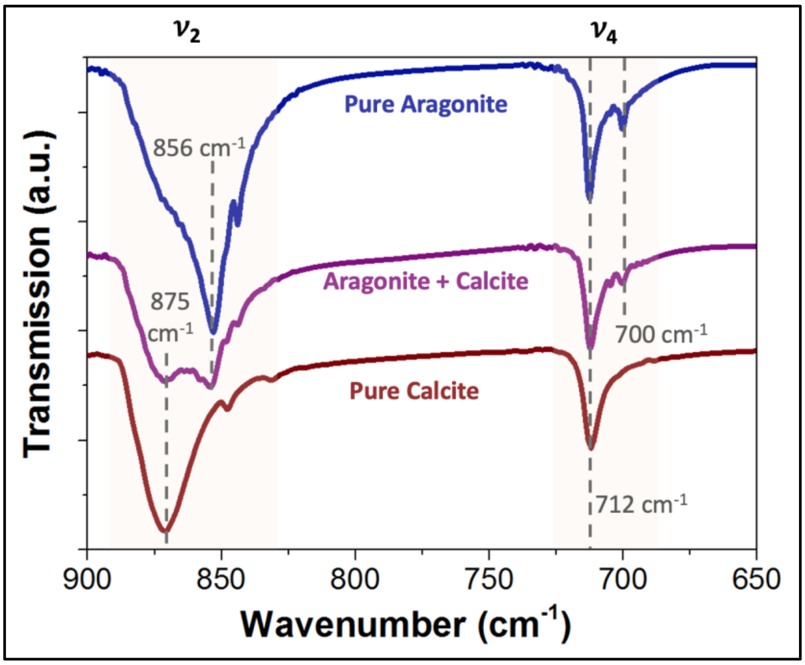

Figure S2. FTIR spectra of $CaCO_3$ precipitated with $[Ca^{2+}]$ = 10 mM and [b-$PEI_{1,200}$] = 10^{-6} – 10^{-3} mg mL^{-1}. A pure calcite phase is precipitated at the lowest polyelectrolyte concentration, as shown by the exclusive presence of ν_2 (out-of-plane CO_3 bending), ν_3 (asymmetric CO_3 stretching) and ν_4 (in-plane CO_3 bending) of calcite (red). The highest b-PEI concentration yielded a pure phase of aragonite, as shown by the presence of the additional ν_1 (symmetric stretching) in the IR spectra (blue). Intermediate b-PEI concentrations generated mixtures of calcite and aragonite (purple), where the characteristics peaks of both polymorphs were recorded in the samples.

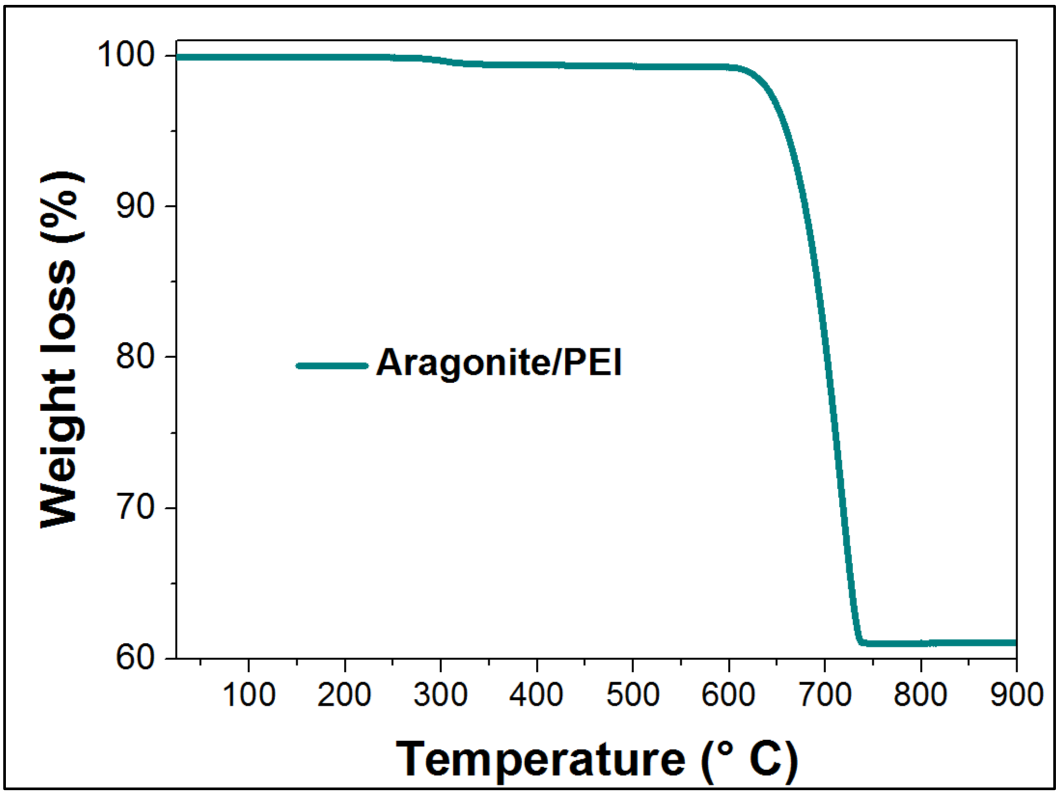

Figure S3. TGA data recorded in air for pure aragonite precipitated at $[Ca^{2+}]$ = 10 mM and 10^{-3} mg mL^{-1} b-PEI (MW = 1,200 g mol^{-1}). Less than 1 wt.% of polymer is incorporated within the aragonitic crystal structure.

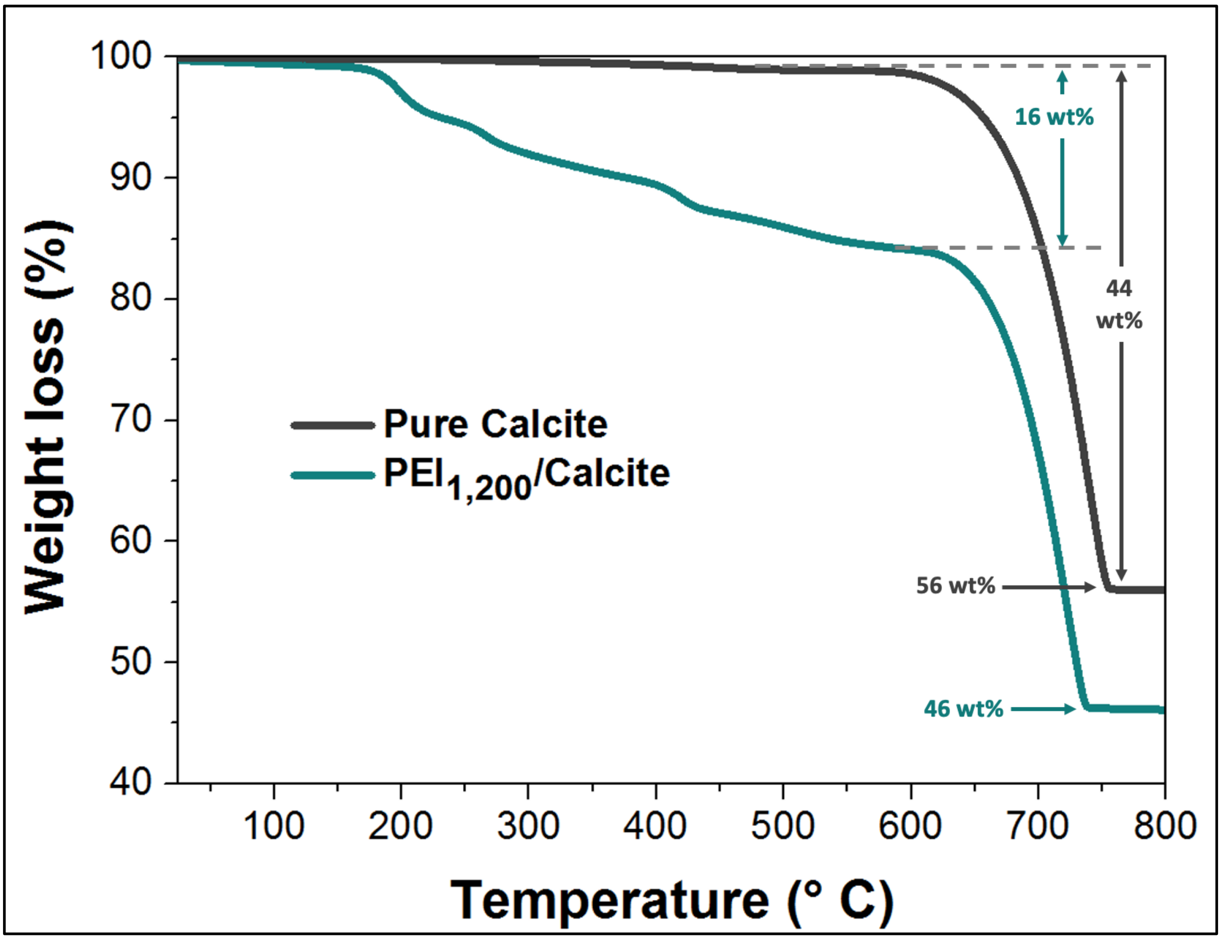

Figure S4. TGA data recorded in air for pure calcite (dark grey) and calcite incorporating b-PEI (MW = 1,200 g mol^{-1}, cyan) grown at [b-$PEI_{1,200}$] = 2.5 mg mL^{-1} and [Ca^{2+}] = 10 mM. The weight loss measured for the PEI/calcite composites between room temperature and ≈ 650°C corresponds to the amount of PEI incorporated within calcite.

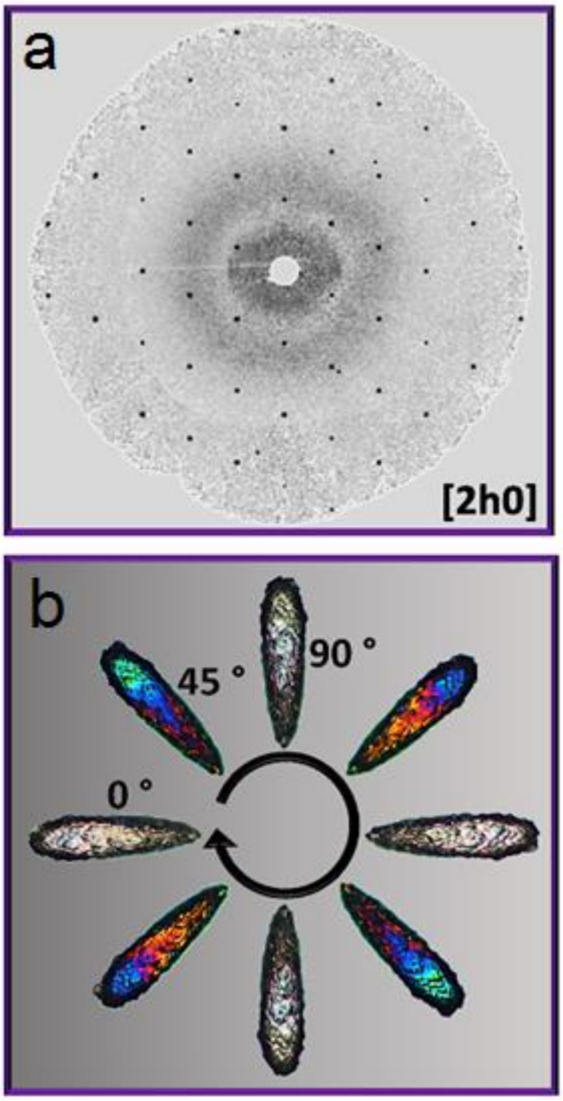

Figure S5. Analysis of a calcite single crystal grown at [b-$PEI_{1,200}$] = 2.5 mg mL^{-1} and [Ca^{2+}] = 10 mM. (a) Single-crystal XRD of a PEI/calcite composite crystal showing that its single-crystal character. (b) Polarized optical micrograph of a PEI/calcite crystal, taken at different angles with respect to the plane of polarized light, showing distinct angles at which, the whole crystal displays bright birefringence.

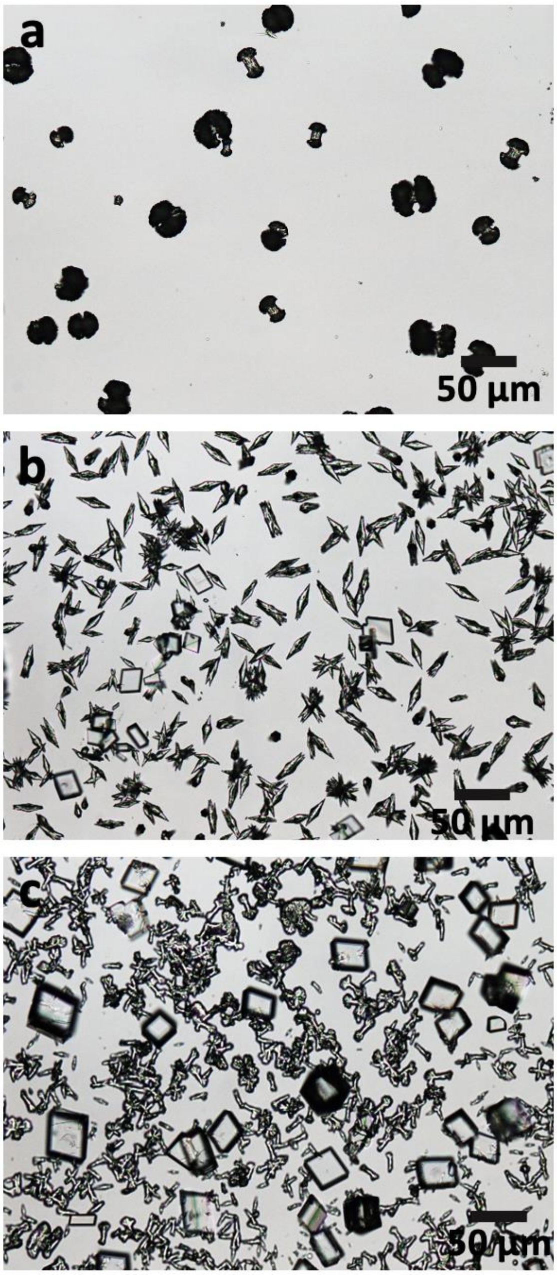

Figure S6. (a-c) Optical images of $CaCO_3$ crystals precipitated at [b-$PEI_{1,200}$] = 10^{-3} mg mL^{-1} at [Ca^{2+}] = 1 – 50 mM. (a) Pure aragonite forms at [Ca^{2+}] = 1 mM, and increasing proportions of calcite precipitated at higher supersaturations, (b) [Ca^{2+}] = 20 mM and (c) [Ca^{2+}] = 50 mM.

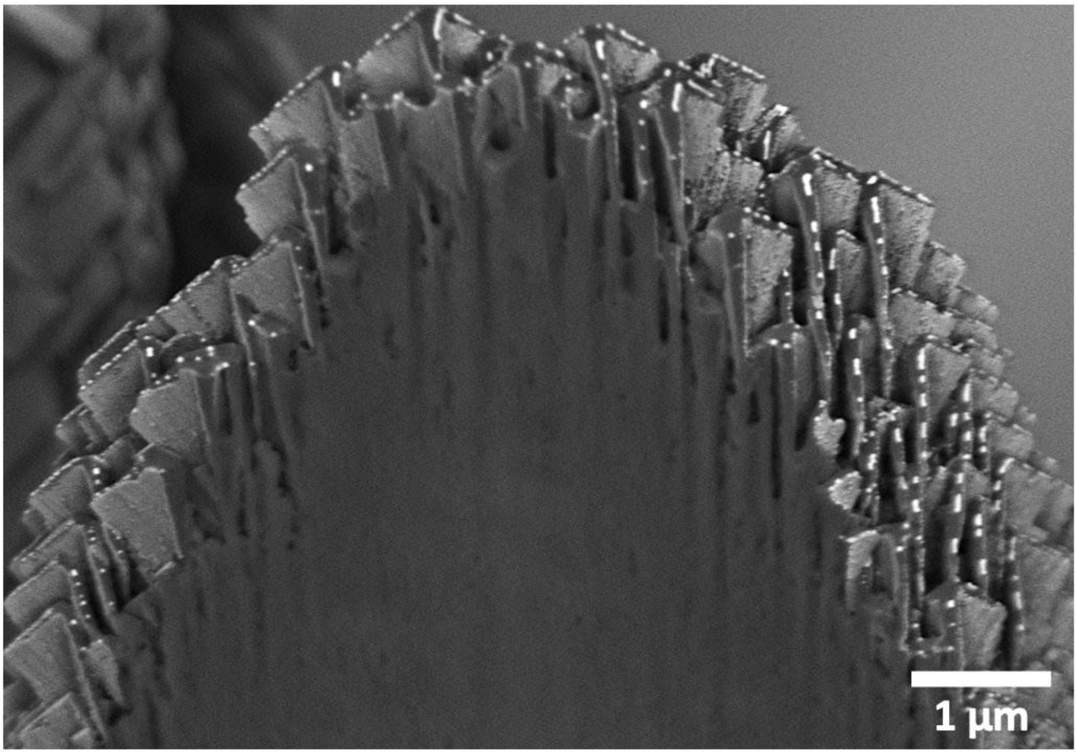

Figure S7. FIB-SEM cross-section of a calcite crystal precipitated at [Ca^{2+}] = 10 mM and [b-PEI] = 1 mg mL^{-1}. The micrograph shows a continuous crystalline core decorated with surface patterns, which are exclusively expressed in the outer ≈ 500 nm layer of the crystal.

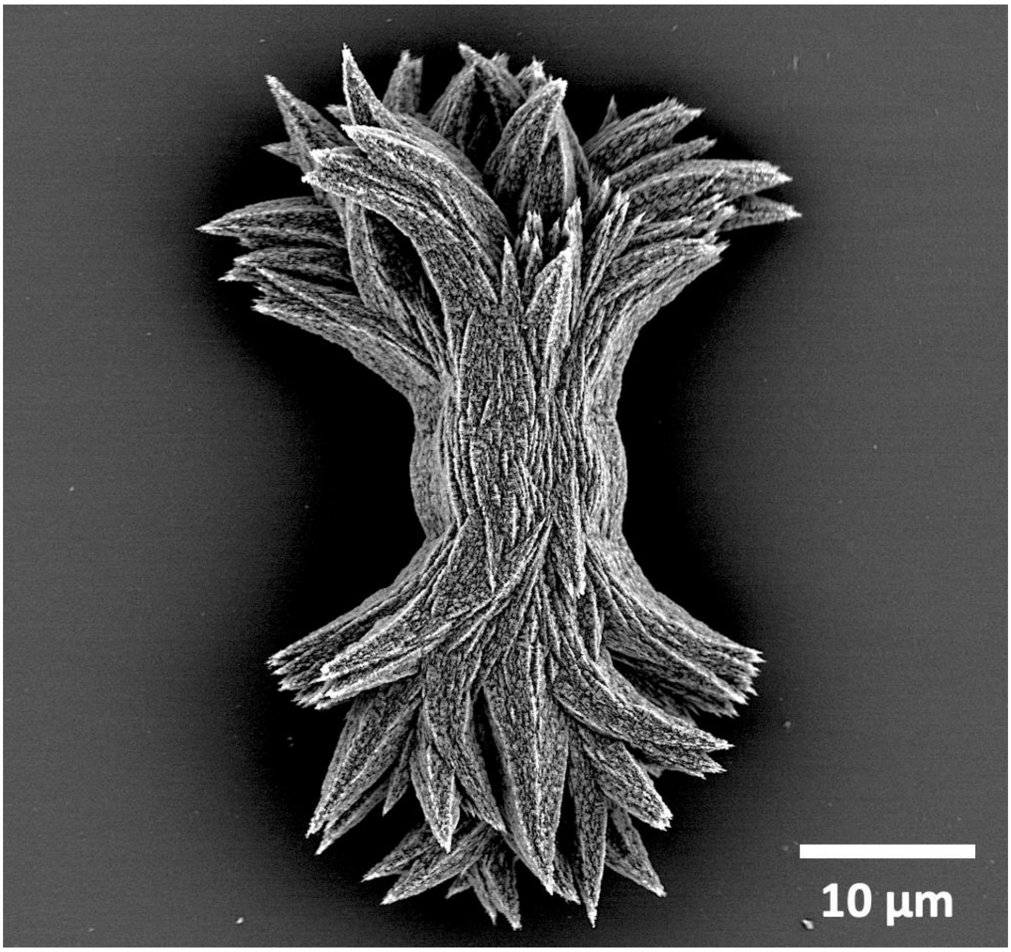

Figure S8. SEM image of mature aragonite crystal precipitated at [Ca^{2+}] = 10 mM and [b-PEI] = 10^{-3} mg mL^{-1}.

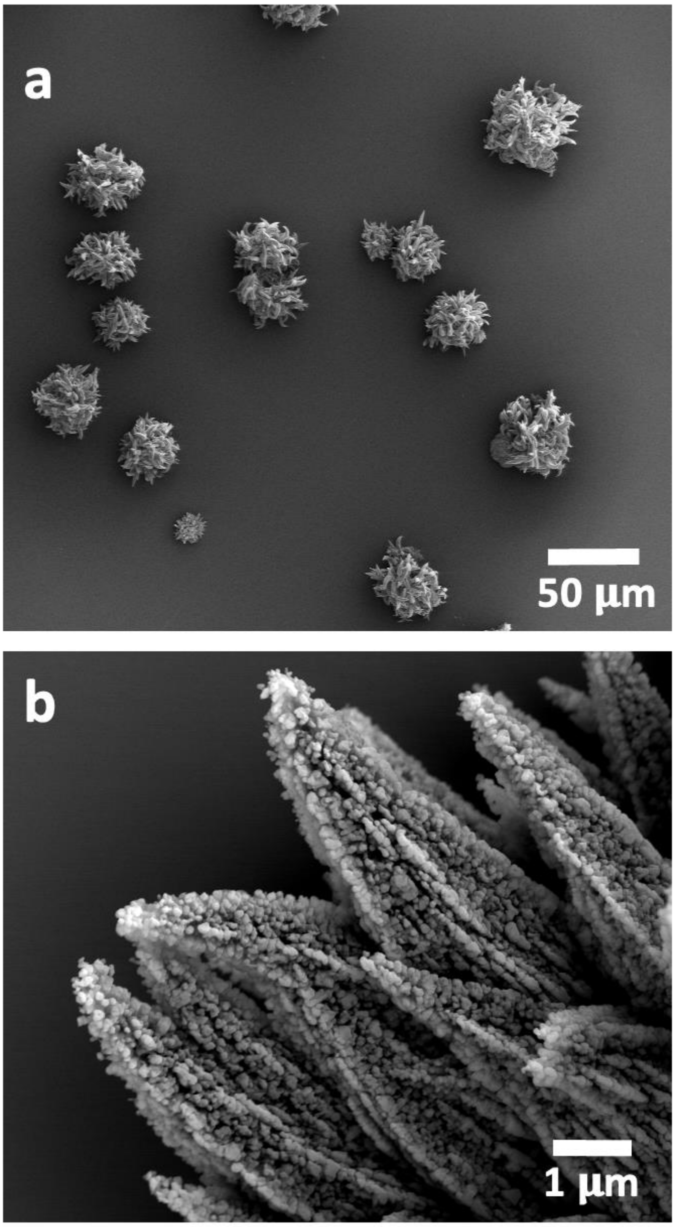

Figure S9. (a-b) SEM micrographs of aragonite crystals formed in the presence of [Ca^{2+}] = 10 mM and 10^{-3} mg mL^{-1} linear PEI (MW = 10,000 g mol^{-1}). (b) The aragonite crystals display a nanogranular texture.

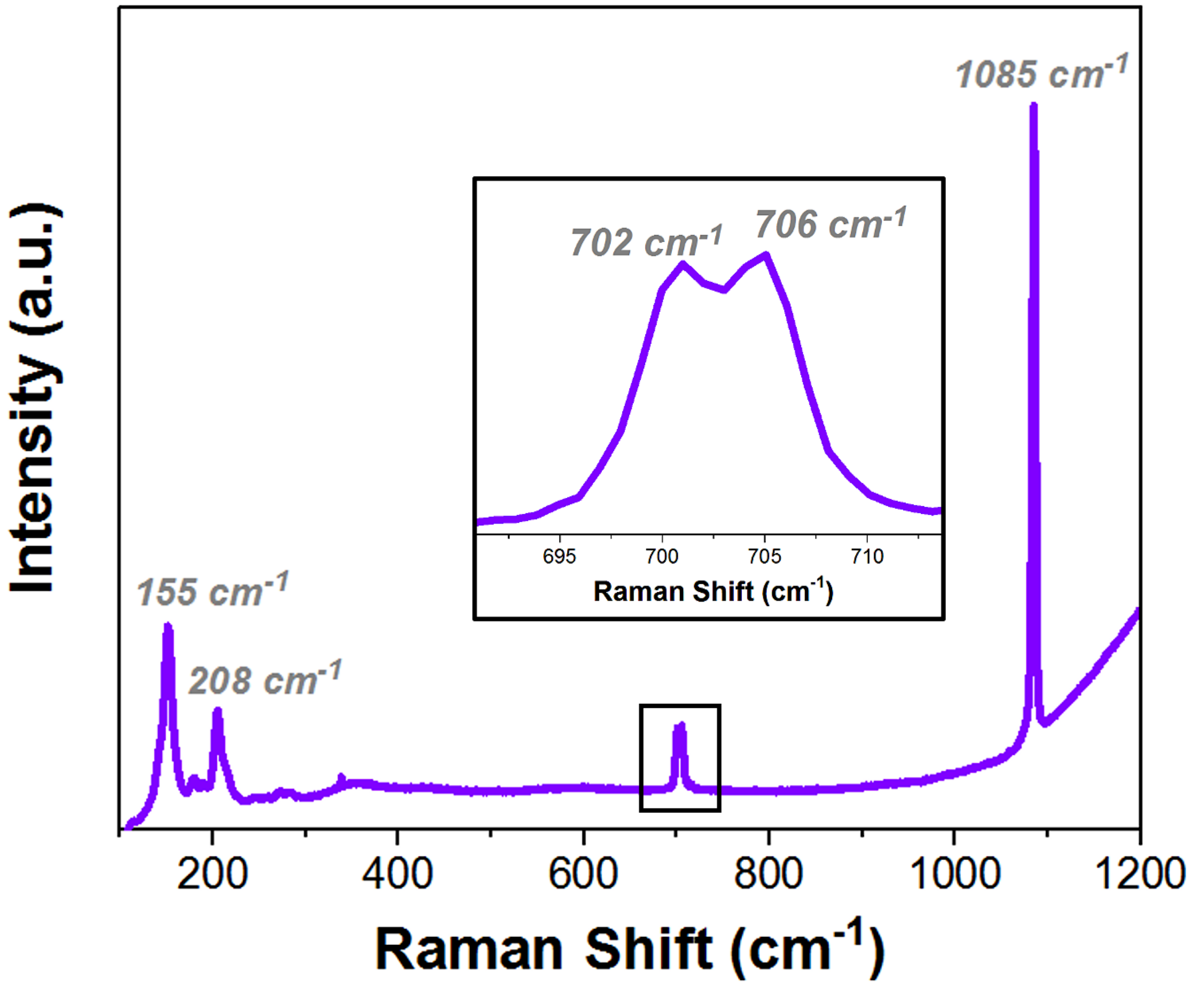

Figure S10. Raman spectrum of $CaCO_3$ crystals precipitated at [Ca^{2+}] = 10 mM in the presence of 10^{-3} mg mL^{-1} linear PEI (MW = 4,000 g mol^{-1}). The characteristics peaks ν_1 (1085 cm^{-1}), ν_4 (702 cm^{-1} and 706 cm^{-1}), and lattice modes (155 cm^{-1} and 208 cm^{-1}) of aragonite are recorded.

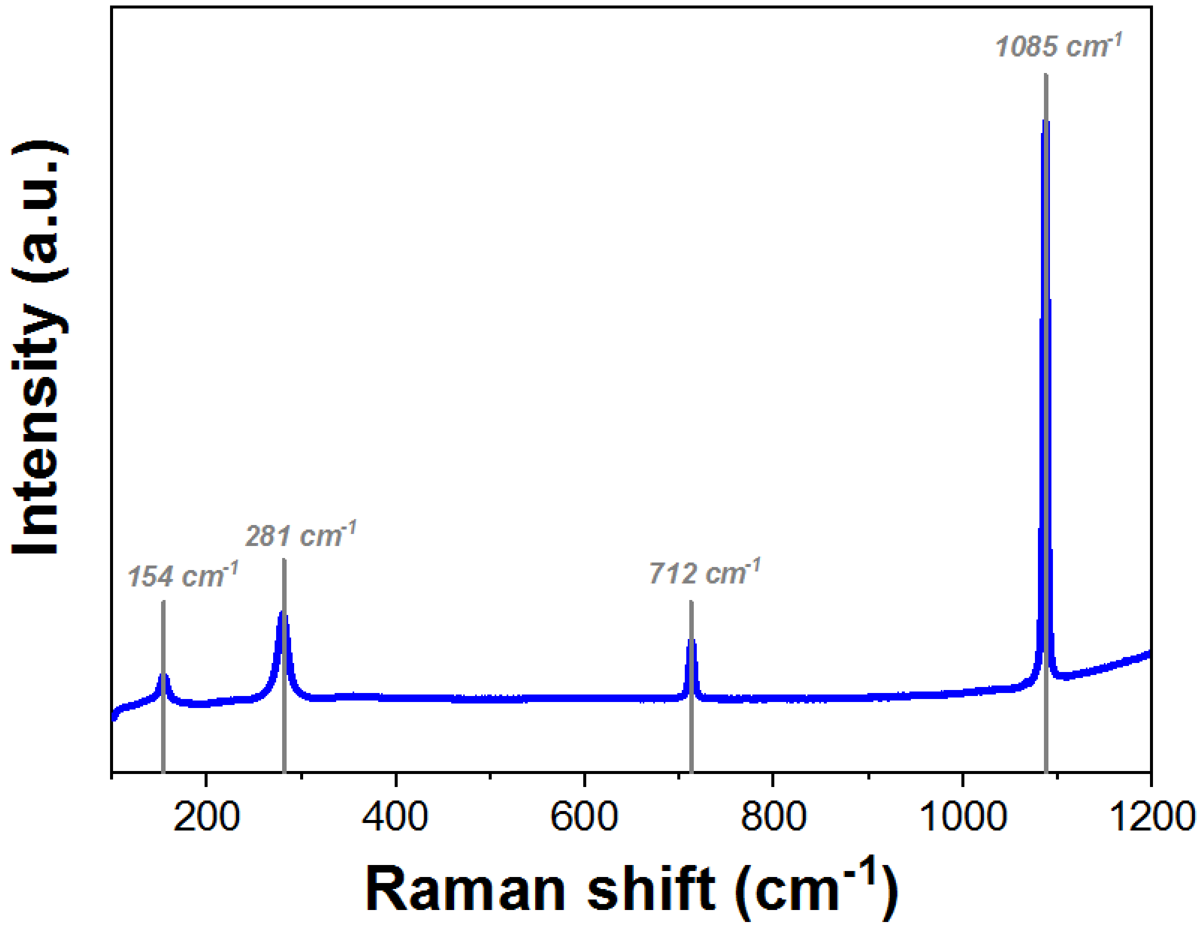

Figure S11. Raman spectrum of $CaCO_3$ crystals precipitated at [Ca^{2+}] = 10 mM and in the presence of 1 mg mL^{-1} linear PEI (MW = 4,000 g mol^{-1}). The characteristics peaks ν_1 (1085 cm^{-1}), ν_4 (712 cm^{-1}), and lattice modes (154 cm^{-1} and 281 cm^{-1}) of calcite are recorded.

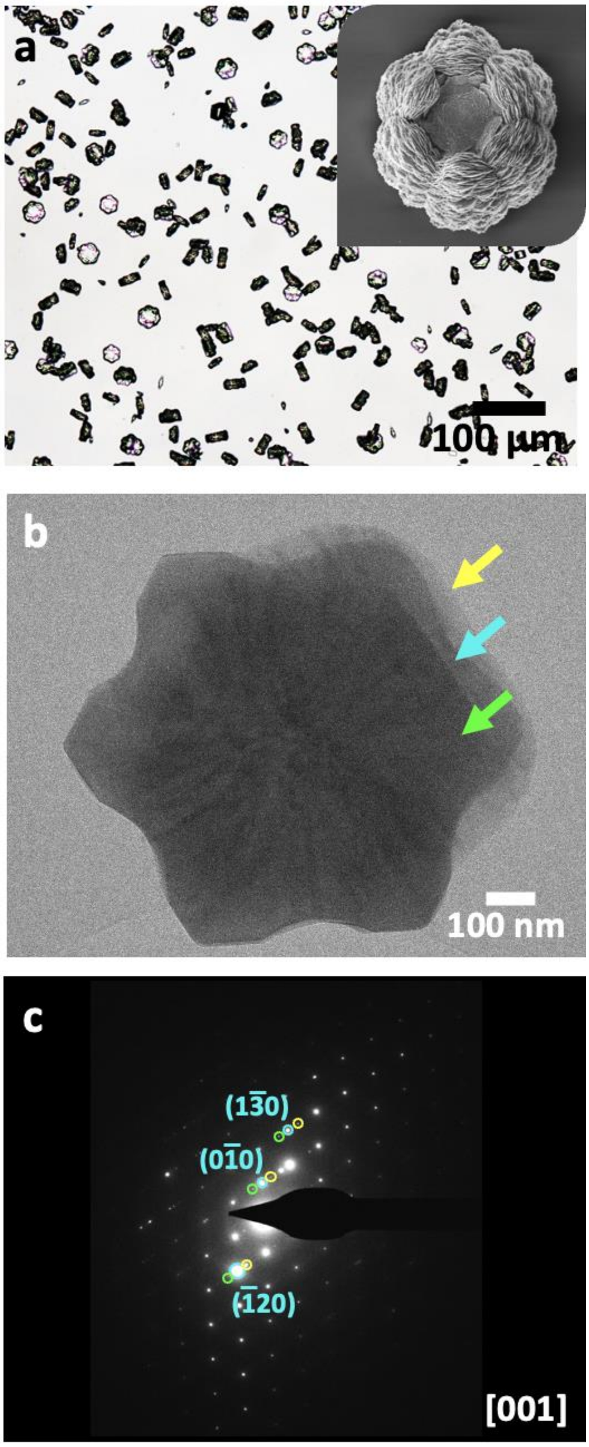

Figure S12. (a) Optical and SEM (inset) images of flower-like vaterite crystals formed at [Ca^{2+}] = 10 mM and 1 mg mL^{-1} of longer linear PEI chains (MW = 10,000 g mol^{-1}). (b) TEM image of an incipient vaterite crystal showing that it consists of single crystals stacked on their (001) faces as demonstrated by (c) overlaid SAED patterns.

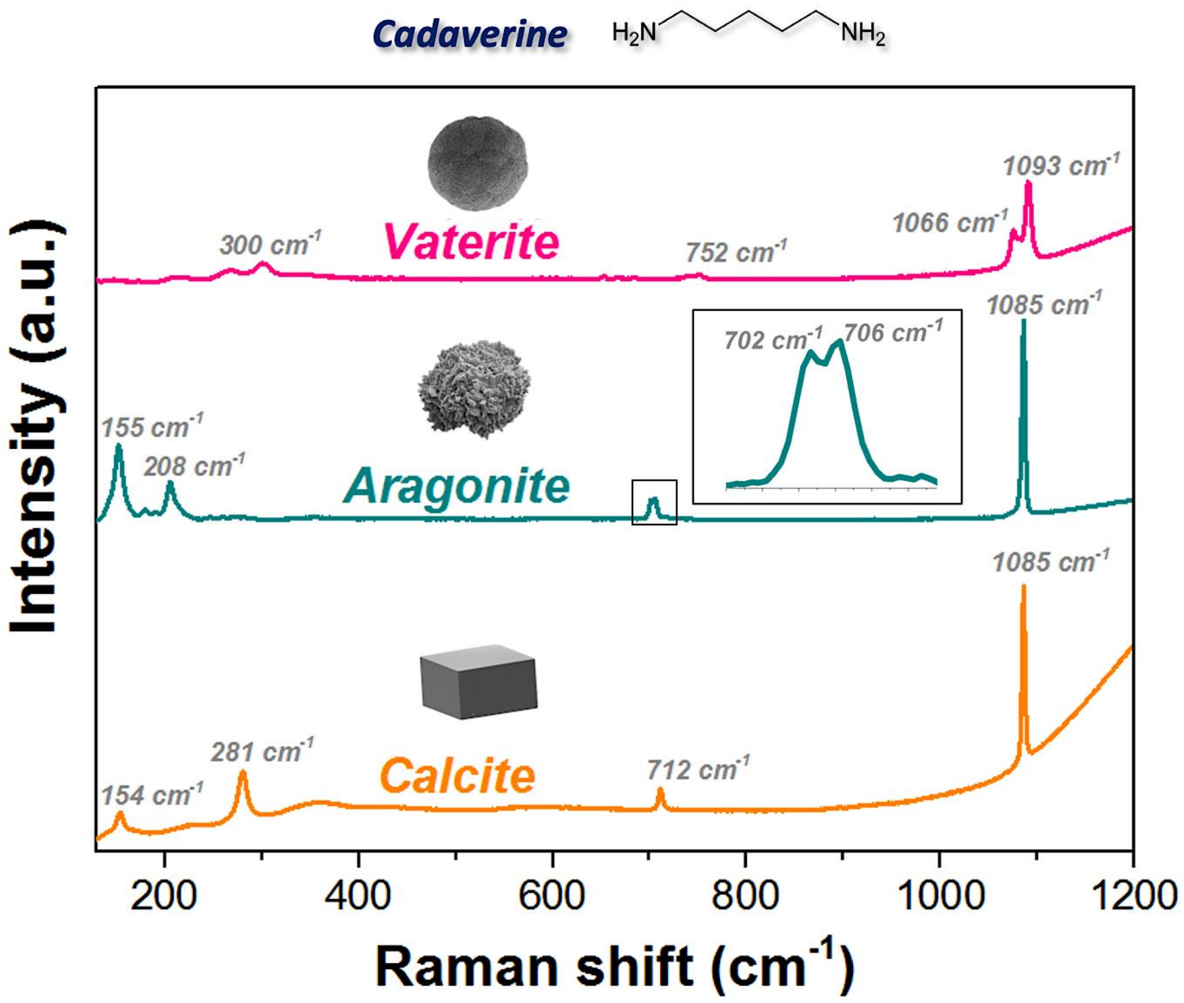

Figure S13. Raman spectra of $CaCO_3$ crystals precipitated in the presence of $[Ca^{2+}]$ = 2.5 mM and 0.05 mg mL^{-1} (orange), 0.1 mg mL^{-1} (cyan) and 0.5 mg mL^{-1} (pink) cadaverine. The characteristics peaks ν_1 (1085 cm^{-1}), ν_4 (712 cm^{-1}), and lattice modes (154 cm^{-1} and 281 cm^{-1}) of calcite; ν_1 (1085 cm^{-1}), ν_4 (702 cm^{-1} and 706 cm^{-1}), and lattice modes (155 cm^{-1} and 208 cm^{-1}) of aragonite; and ν_1 (1066 cm^{-1} and 1093 cm^{-1}), ν_4 (752 cm^{-1}), and lattice mode (300 cm^{-1}) of vaterite are recorded.

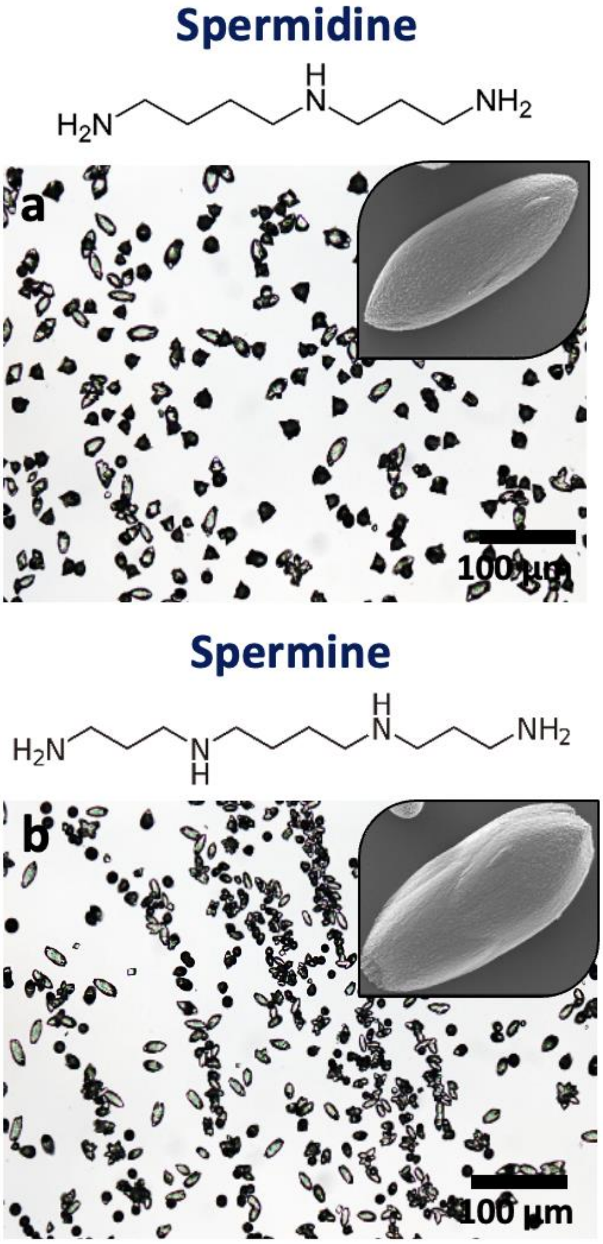

Figure S14. Optical and SEM (inset) images of *c*-axis elongated, and rough calcite crystals precipitated at [Ca^{2+}] = 10 mM and (a) 0.5 mg mL^{-1} spermidine, and (b) 0.5 mg mL^{-1} spermine.

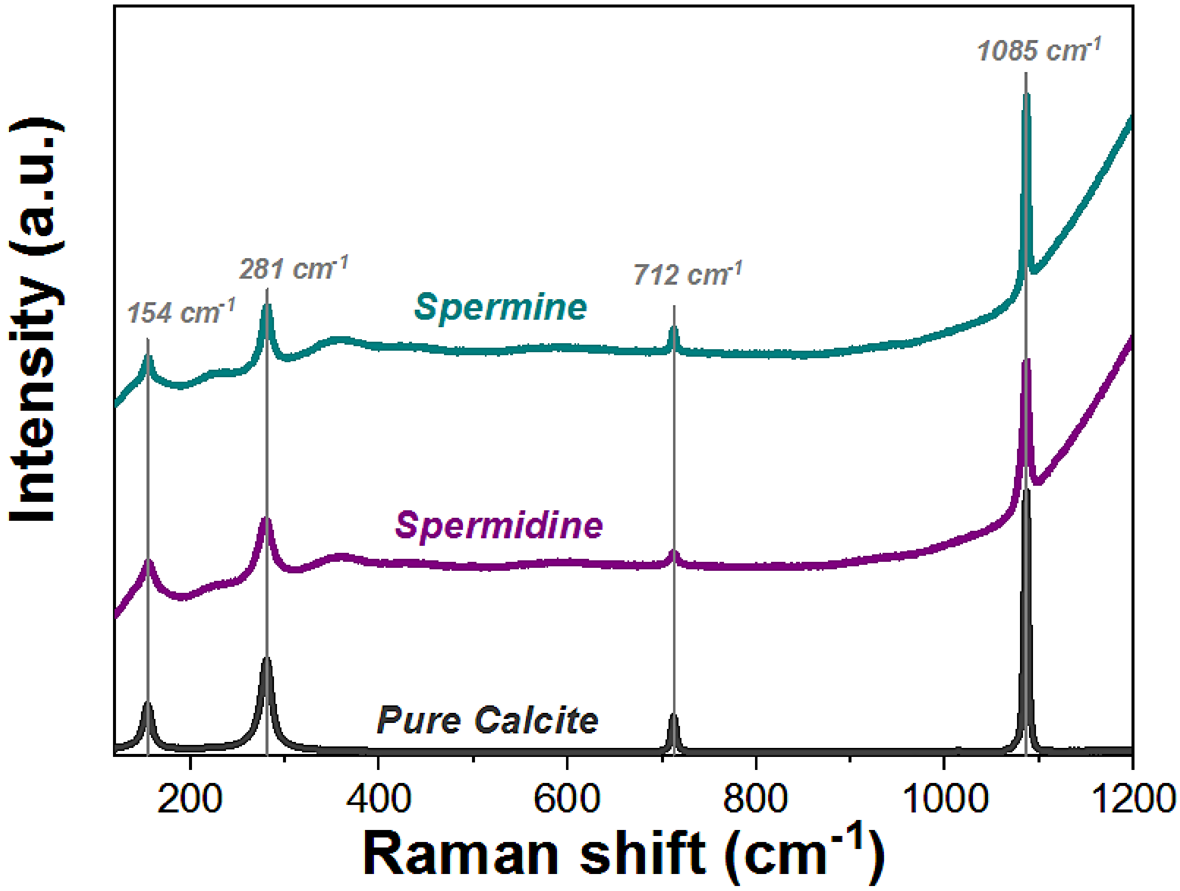

Figure S15. Raman spectra of $CaCO_3$ crystals precipitated at [Ca^{2+}] = 10 mM and in the presence of 0.5 mg mL^{-1} spermidine (purple) or spermine (cyan) in solution. The characteristics peaks: ν_1 (1085 cm^{-1}), ν_4 (712 cm^{-1}), and lattice modes (154 cm^{-1} and 281 cm^{-1}) of calcite (dark grey) are recorded.

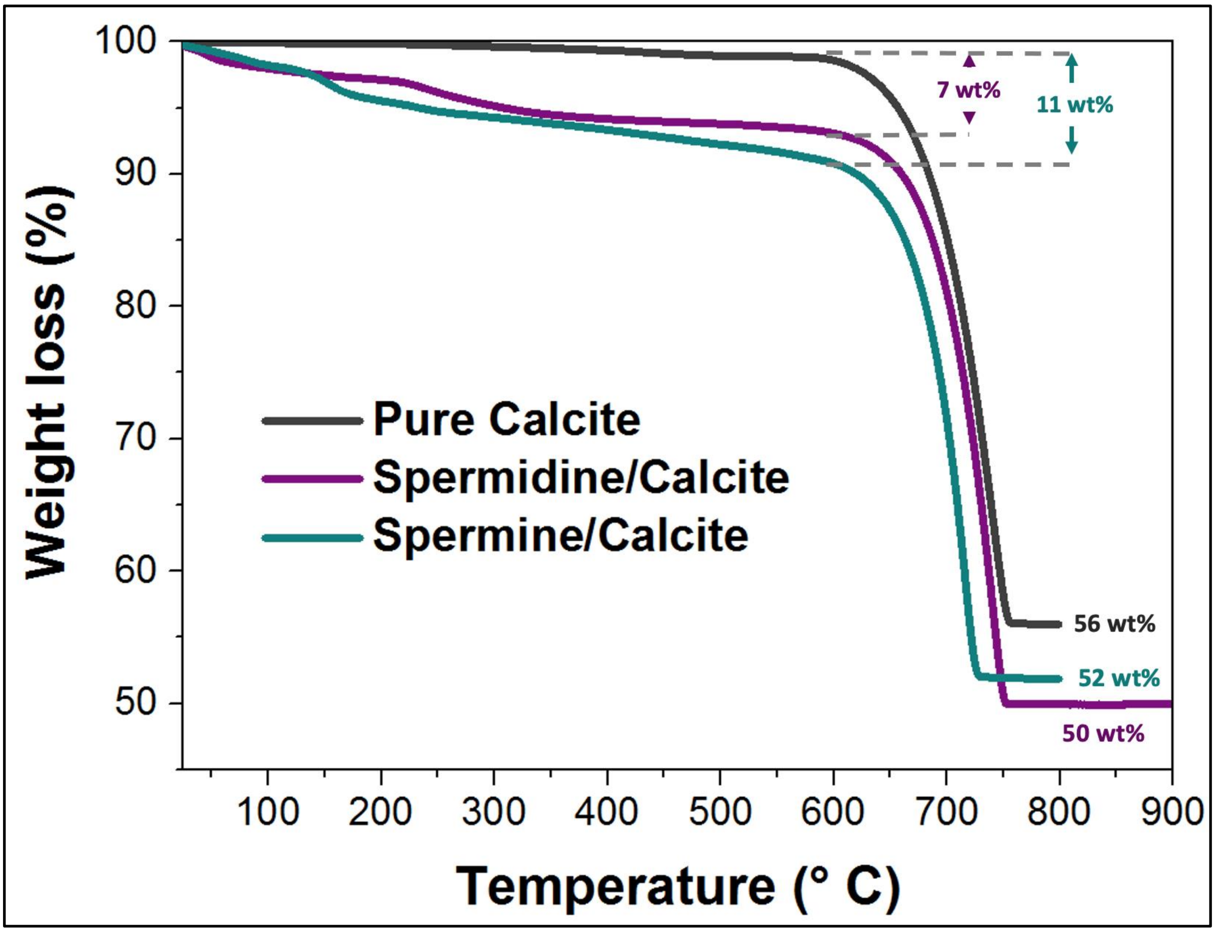

Figure S16. TGA data recorded in air for pure calcite (dark grey), and calcite incorporating spermidine (purple), and spermine (cyan). The weight losses between room temperature and 600 °C correspond to the amounts of biogenic amines incorporated within calcite that are thermally decomposed.

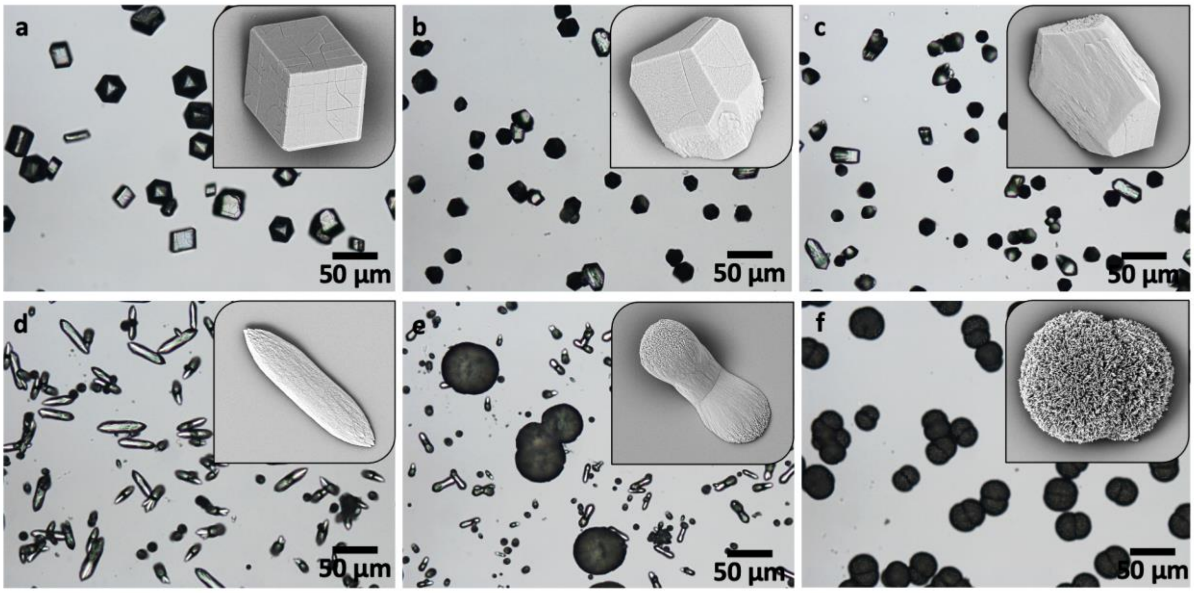

Figure S17. Optical and SEM images (insets) of $CaCO_3$ crystals precipitated with [Ca^{2+}] = 10 mM and (a) [Mg^{2+}] = 2.5 mM, (b) [Mg^{2+}] = 7.5 mM, (c) [Mg^{2+}] = 15 mM, (d) [Mg^{2+}] = 25 mM, (e) [Mg^{2+}] = 35 mM, and (f) [Mg^{2+}] = 50 mM. Increase in magnesium ions in solution generates calcite crystals that are gradually smaller and more elongated along their c-axes in the regime [Mg^{2+}]/[Ca^{2+}] < 5, while increasing amounts of aragonite crystals formed and a pure phase was generated at [Mg^{2+}]/[Ca^{2+}] = 5.

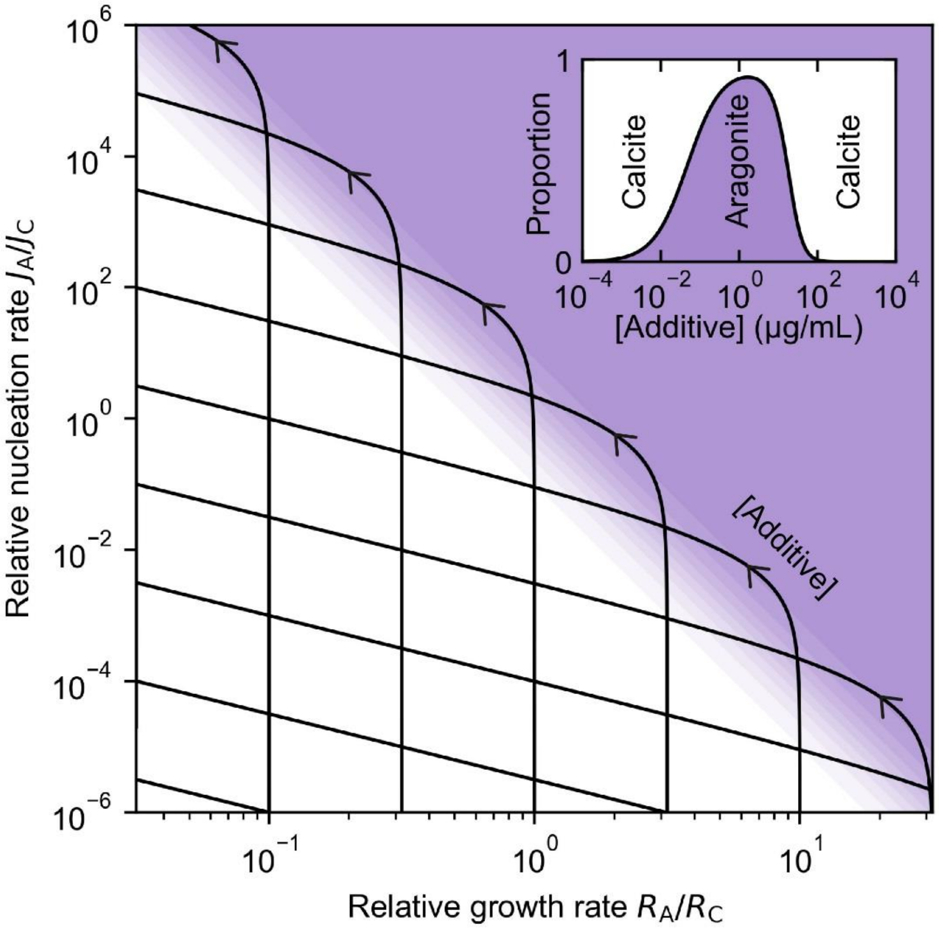

Figure S18. Theoretical model capturing the competition between calcite (C) and aragonite (A). Nucleation rates are termed J_i and normal growth rates R_i. The purple and white colors represent a high abundance of aragonite and calcite, respectively. The model yields a family of solutions as there is insufficient data to single out one curve. Moving along any one of the curves in the direction of the arrow corresponds to an increase in the polyamine concentration *c* and produces an increase in the nucleation rate of aragonite followed by an inhibition of its growth. This two-step process sees the aragonite come to dominate only to be smothered back out of existence, as shown in the inset.

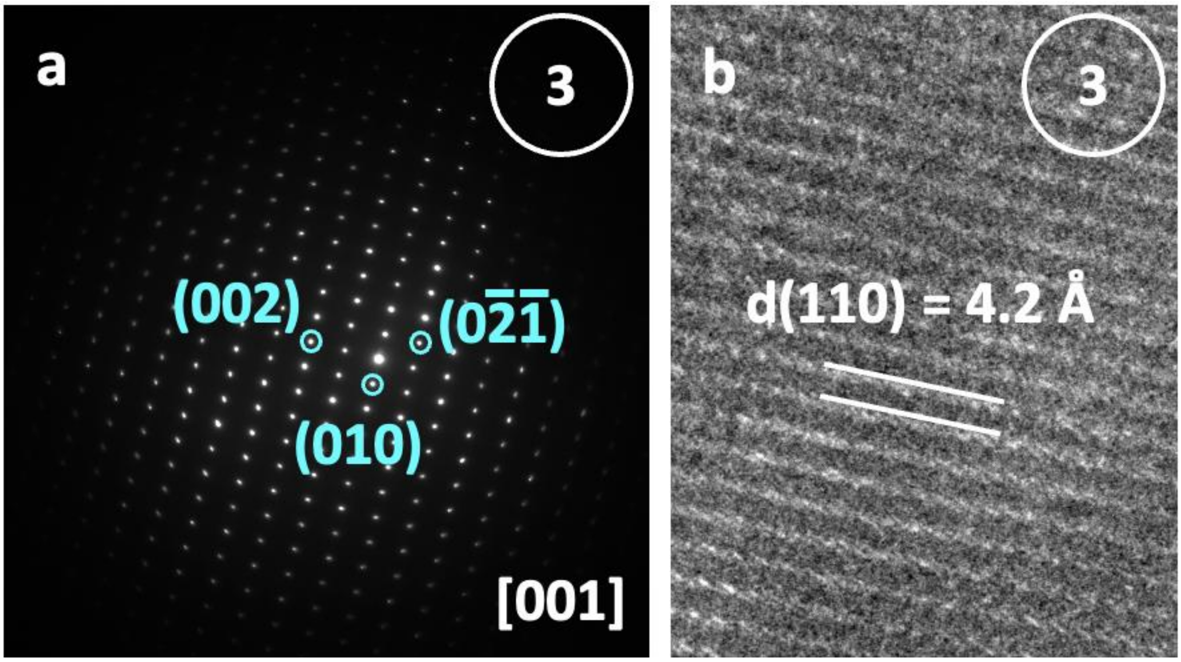

Figure S18. (a) SAED pattern showing that mature aragonite diffracts as a perfect single crystal. (b) HRTEM image of mature aragonite showing continuous lattice fringes indicative of single-crystal character.

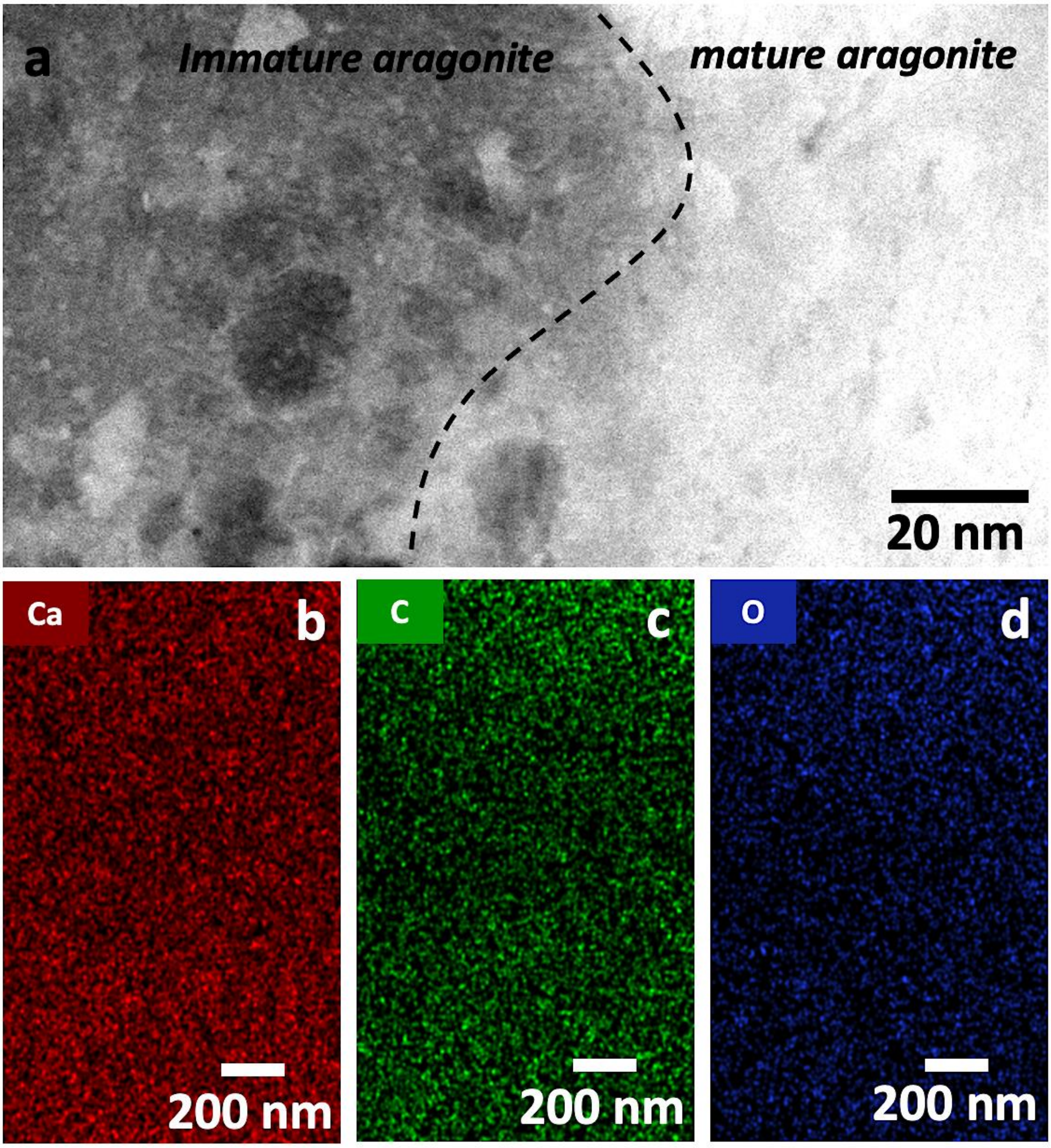

Figure S19. (a) HAADF-STEM image of the internal structure of an aragonite crystal precipitated at [Ca^{2+}] = 10 mM and [b-PEI (MW = 1,200 g mol^{-1})] = 10^{-3} mg mL^{-1} and (b-d) STEM-EDX maps showing the uniform distribution of $CaCO_3$ throughout the sample.
